# Enhanced detoxification of Cr^6+^ by *Shewanella oneidensis via* adsorption on spherical and flower-like manganese ferrite nanostructures†

**DOI:** 10.1039/d2na00691j

**Published:** 2023-01-16

**Authors:** Diana S. Raie, Ioannis Tsonas, Melisa Canales, Stefanos Mourdikoudis, Konstantinos Simeonidis, Antonis Makridis, Dimitrios Karfaridis, Shanom Ali, Georgios Vourlias, Peter Wilson, Laurent Bozec, Lena Ciric, Nguyen Thi Kim Thanh

**Affiliations:** a Biophysics Group, Department of Physics and Astronomy, University College London Gower Street London WC1E 6BT UK ntk.thanh@ucl.ac.uk http://www.ntk-thanh.co.uk; b UCL Healthcare Biomagnetics and Nanomaterials Laboratories 21 Albemarle Street London W1S 4BS UK; c UCL Electronic and Electrical Engineering, UCL Gower Street London WC1E 7JE UK; d Healthy Infrastructure Research Group, Department of Civil, Environmental & Geomatic Engineering, UCL Gower Street London WC1E 6BT UK; e Department of Physics, Aristotle University of Thessaloniki 54124 Thessaloniki Greece; f Environmental Research Laboratory, ClinicalMicrobiology and Virology, University College London Hospitals NHS Foundation Trust London UK; g Faculty of Dentistry, University of Toronto Toronto Ontario Canada

## Abstract

Maximizing the safe removal of hexavalent chromium (Cr^6+^) from waste streams is an increasing demand due to the environmental, economic and health benefits. The integrated adsorption and bio-reduction method can be applied for the elimination of the highly toxic Cr^6+^ and its detoxification. This work describes a synthetic method for achieving the best chemical composition of spherical and flower-like manganese ferrite (Mn_*x*_Fe_3−*x*_O_4_) nanostructures (NS) for Cr^6+^ adsorption. We selected NS with the highest adsorption performance to study its efficiency in the extracellular reduction of Cr^6+^ into a trivalent state (Cr^3+^) by *Shewanella oneidensis* (*S. oneidensis*) MR-1. Mn_*x*_Fe_3−*x*_O_4_ NS were prepared by a polyol solvothermal synthesis process. They were characterised by powder X-ray diffraction (XRD), transmission electron microscopy (TEM), X-ray photoelectron spectrometry (XPS), dynamic light scattering (DLS) and Fourier transform-infrared (FTIR) spectroscopy. The elemental composition of Mn_*x*_Fe_3−*x*_O_4_ was evaluated by inductively coupled plasma atomic emission spectroscopy. Our results reveal that the oxidation state of the manganese precursor significantly affects the Cr^6+^ adsorption efficiency of Mn_*x*_Fe_3−*x*_O_4_ NS. The best adsorption capacity for Cr^6+^ is 16.8 ± 1.6 mg Cr^6+^/g by the spherical Mn_0.2_^2+^Fe_2.8_^3+^O_4_ nanoparticles at pH 7, which is 1.4 times higher than that of Mn_0.8_Fe_2.2_O_4_ nanoflowers. This was attributed to the relative excess of divalent manganese in Mn_0.2_^2+^Fe_2.8_^3+^O_4_ based on our XPS analysis. The lethal concentration of Cr^6+^ for *S. oneidensis* MR-1 was 60 mg L^−1^ (determined by flow cytometry). The addition of Mn_0.2_^2+^Fe_2.8_^3+^O_4_ nanoparticles to *S. oneidensis* MR-1 enhanced the bio-reduction of Cr^6+^ 2.66 times compared to the presence of the bacteria alone. This work provides a cost-effective method for the removal of Cr^6+^ with a minimum amount of sludge production.

## Abstract 2

An enhancement strategy for the bio-reduction of Cr^6+^ in the presence of manganese ferrite nanostructures using *Shewanella oneidensis.*

## Graphical abstract

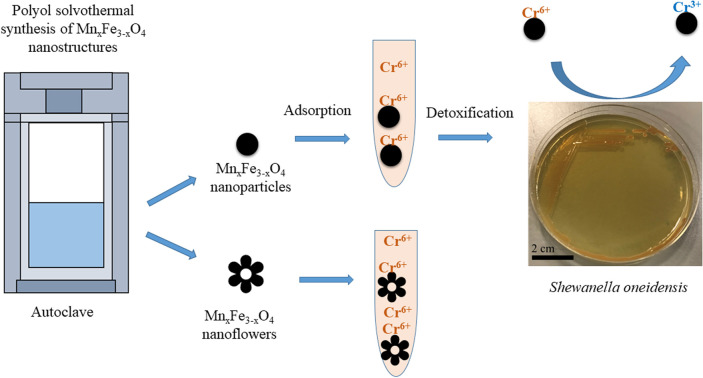

## 1. Introduction

Chromium (Cr) is a common environmental pollutant coming from several industries such as wood preservation,^1^ leather tanning,^2^ steel production,^3,4^ wool dyeing,^5^ painting,^6^ refractories,^4^ lasers,^7^ and electroplating,^8^ among others. End-of-life products such as unwanted steel, wood,^1^ leather and textiles, among other materials are extra sources of Cr leakage in the environment. The release of Cr in the environment was also attributed to mining activities,^9^ and improper waste treatment associated with industrial processes.^10^ Various Cr-bearing minerals, including chromite, are available in the soil, and bedrock also releases natural Cr into the environment.^10^ It mainly occurs in two valence states, which are highly toxic carcinogenic^11^ Cr^6+^ and less toxic Cr^3+^. Various technologies have been developed to tackle the presence of Cr^6+^, including membranes,^12^ coagulation,^13^ photocatalysis,^14^ electrochemical treatments,^13^ adsorption^15,16^ and biological treatments.^17,18^ Integrating both adsorption and biological reduction of Cr^6+^ together has been proposed as a promising solution.^19^ Applying such combined methods can overcome the accessibility of certain technologies,^20^ using less toxic chemicals and reducing the production of contaminated toxic waste.^19^ The recovered chromium can be used in metallurgical industries and minimize the contaminated landfill.^21,22^

Microbial reduction of Cr^6+^ has been regarded as a suitable Cr remediation approach because of being more eco-friendly than the conventional physico-chemical strategies, which are often costly. Recently, many types of bacteria have been reported to detoxify Cr^6+^ to less toxic Cr^3+^, including dissimilatory metal-reducing bacteria such as *Shewanella oneidensis* MR-1.^23,24^ Under anaerobic conditions, *S. oneidensis* can use Cr^6+^ as a terminal electron acceptor,^25^ however cells exposed to Cr^6+^ exhibited a loss in their enzymatic activity and cell lysis.^26^ The bactericidal concentration of Cr^6+^ was reported to be ∼42–65 mg L^−1^ for *S. oneidensis* MR-1.^26,27^ A lethal effect of heavy metals on the microbes during respiration^26,29–31^ was considered as a potential limitation for the bio-remediation of Cr^6+^.^26^ Compared with physical and chemical materials, the concentration of Cr^6+^ that can be reduced by bacteria is much lower, and it is a great challenge to improve the efficiency of bioremediation.^28^

Enhancing the bacterial tolerance to Cr^6+^ is an effective way to improve the reduction of Cr^6+^. Zero-valent iron nanoparticles (ZVI NPs) can easily be oxidised to ferric oxides and hydroxides in water. The active surface of ZVI NPs can be decreased due to the attached layers of iron oxides and hydroxides. *Shewanella*, as iron-reducing bacteria, can reduce the adsorbed Fe^3+^ to Fe^2+^, which reverses the oxidation of ZVI NPs, as shown in a review by Dong *et al.*^32^ Hematite (α-Fe_2_O_3_) particles enhanced the bio-reduction of Cr^6+^ bio-reduction by *S. oneidensis* MR-1, but they cause cytotoxicity to such kind of bacteria.^33^ The reduction of Cr^6+^ by *S. oneidensis* was enhanced by goethite (α-FeOOH) and humic acid through the bio-reduction of Fe^3+^ to Fe^2+^. The reactivity of magnetite (Fe_3_O_4_) was increased by microbial Fe^3+^ reduction to form Fe^2+^, which then can reduce Cr^6+^.^34,35^

A biocompatible material such as manganese ferrite (MnFe_2_O_4_)^36^ was considered for enhancing microbial respiration of Cr^6+^. This ferrite was used to accelerate extracellular electron transfer in the microbial fuel cell,^37,38^ and it showed the highest adsorption capacity among other ferrites for Cr^6+^.^39^ The maximum adsorption capacity of MnFe_2_O_4_ NPs for Cr^6+^ was reported to be ranging from 31 to 35 mg g^−1^.^36,39,40^

The influence of structural features of Mn_*x*_Fe_3−*x*_O_4_ NPs on Cr^6+^ adsorption has not been thoroughly explored. The effect of the oxidation state of Mn precursors on the chemical structure, morphological and magnetic properties of Mn_*x*_Fe_3−*x*_O_4_ NPs prepared by scalable polyol solvothermal method has been studied in a few reports^41,42^ but not in relation to their adsorption efficiency for heavy metals.

Herein, we report syntheses and characterization of the most suitable chemical structure of Mn_*x*_Fe_3−*x*_O_4_ NPs and nanoflowers (NFs) for the best adsorption capacity of Cr^6+^. The impact of the oxidation states of Mn precursors and variation in Mn doping levels on the chemical structural and morphological characteristics of MnFe_2_O_4_ NPs prepared by polyol solvothermal route has been investigated. The nature of Cr^6+^ adsorption by doped and undoped ferrite NPs and, subsequently, the bio-detoxification of Cr^6+^ by *S. oneidensis* have been studied.

## 2. Results and discussion

### 2.1 Synthesis of nanomaterials

In polyol synthesis, metal precursors are reduced at a high temperature by alcohols (polyols), which can act as a capping agent, solvent and reductant. Then metal nuclei form, grow and controllably coalesce together to produce the desired particles.^43^ In such a non-aqueous solvent, the metal oxide NPs were proposed to be formed *via* two steps. In the first step, solvolysis of the precursor involved an interaction between tetraethylene glycol (TEG) and the selected metal acetylacetonate, causing the generation of metal carboxylate.^44^ The second step is a condensation reaction in which carboxylate reacts with iron leading to the formation of an oxo-bridge between metal (metal–oxygen–metal clusters) and ultimately resulting in the formation of metal oxide nanocrystals.^44^

### 2.2 Characterization of Mn_*x*_Fe_3−*x*_O_4_ NPs

#### 2.2.1 Morphology of Mn_*x*_Fe_3−*x*_O_4_ NPs

**Fig. 1:**
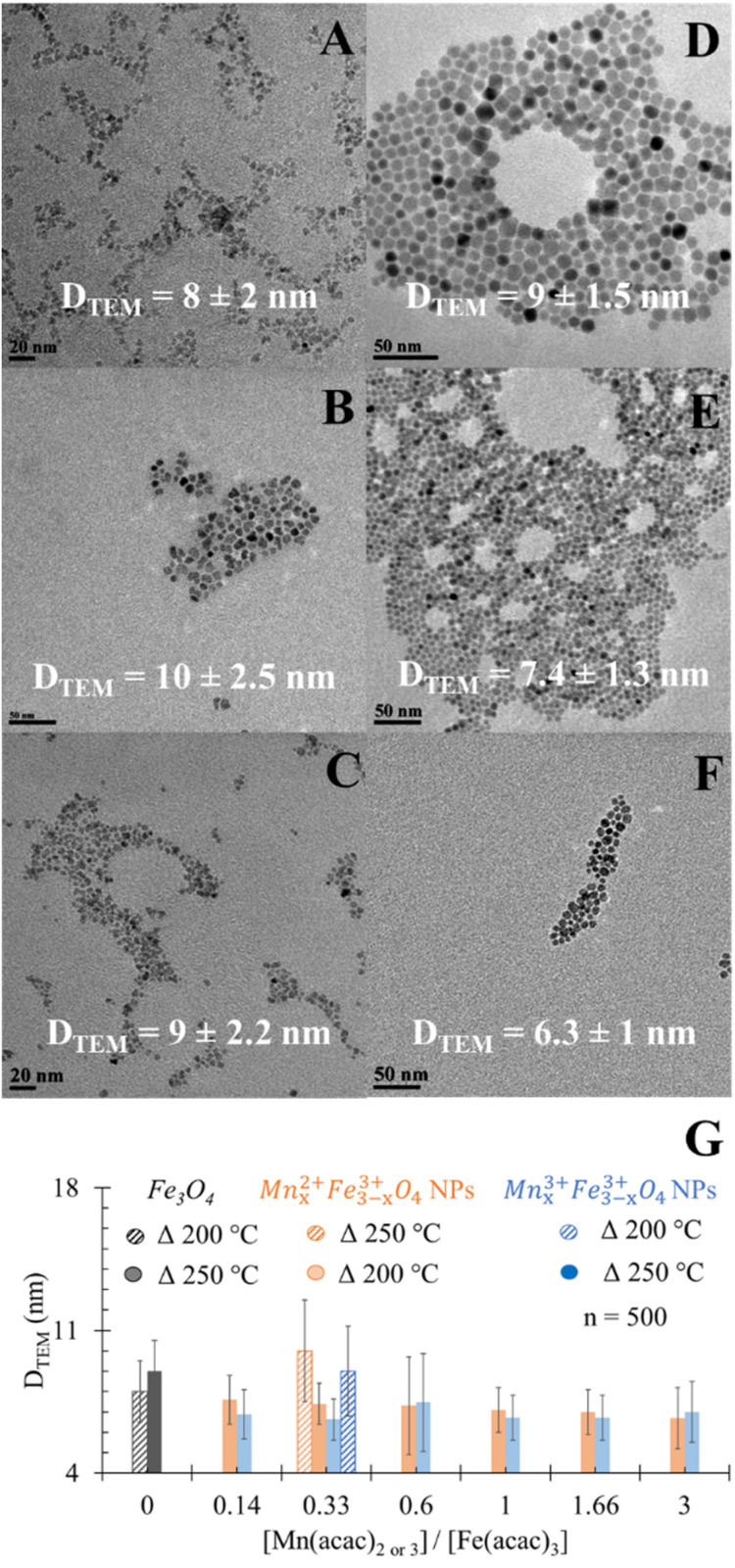
TEM images for the spherical (A) undoped Fe_3_O_4_ NPs, (B) Mn_*x*_Fe_3−*x*_O_4_ of precursor ratio [Mn(acac)_2_]/[Fe(acac)_3_] = 0.33, (C) [Mn(acac)_3_]/[Fe(acac)_3_] = 0.33 prepared at 200 °C as reaction temperatures, (D) undoped Fe_3_O_4_ NPs and (E) and (F) Mn_*x*_Fe_3−*x*_O_4_ NPs prepared with the same precursor ratio but at 250 °C. (G) Impact of reaction temperatures on *D*_TEM_ of Mn_*x*_Fe_3−*x*_O_4_ NPs prepared from [Mn(acac)_2_]/[Fe(acac)_3_] = 0.33. *P < 0.05.

The prepared Mn_*x*_Fe_3−*x*_O_4_ NPs using precursor ratios 0.14 ≤ [Mn(acac)_2 or 3_]/[Fe(acac)_3_] ≤ 3 have nearly spherical shape and are well dispersed on TEM grids, with sufficient interparticle distances as shown in Fig. 1A–C and S1.†*D*_TEM_ (particle diameter determined by TEM) ranged from 5 to 12.5 nm with polydispersity indexes between 0.14 and 0.21, except for 0.33 and 0.6 ratios of [Mn(acac)_3_]/[Fe(acac)_3_], prepared at 200 °C and 250 °C, respectively (see Fig. S2†).

Doping Mn had astatistically insignificant change in the *D*_TEM_ of Mn_*x*_Fe_3−*x*_O_4_ NPs compared to undoped Fe_3_O_4_ NPs (Fig. 1D). In the case of using the divalent Mn precursor, a statistically insignificant change in *D*_TEM_ was observed when increasing the ratio of precursors. This is in agreement with what was reported by Garcia-Soriano *et al.*^45^

#### 2.2.2 Crystal structure of Mn_*x*_Fe_3−*x*_O_4_ NPs

**Fig. 2:**
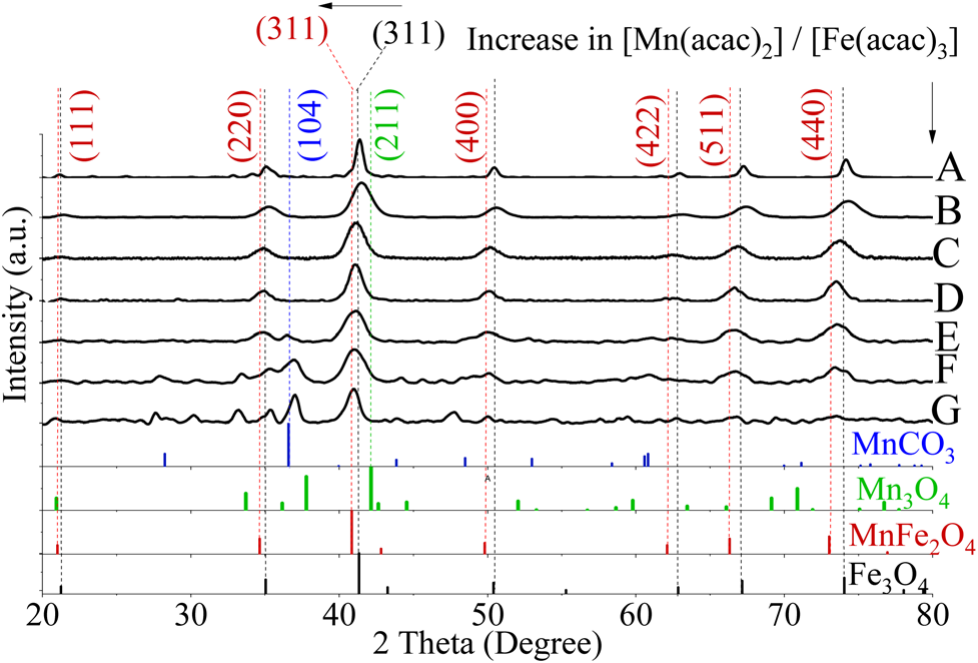
XRD patterns for fcc lattice of Fe_3_O_4_ (A) and Mn_*x*_Fe_3−*x*_O_4_ NPs where [Mn(acac)_2_]/[Fe(acac)_3_] were 0.14 (B), 0.33 (C), 0.6 (D), 1 (E), 1.66 (F), 3 (G). The horizontal arrow pointed out the shifting in the peak of 311 from the reference Fe_3_O_4_ (PDF card no. 01-089-0688) towards the lower diffraction angle of MnFe_2_O_4_ (PDF card no. 00-010-0319) in response to the increase in [Mn(acac)_2_]/[Fe(acac)_3_]. A secondary phase of MnCO_3_ (Reference ICDD PDF card no. 00-044-1472) was found for NPs prepared from (1 ≤ [Mn(acac)_2 or 3_]/[Fe(acac)_3_] ≤ 3) (E)–(G). The synthesis temperature for all NPs was 250 °C. The vertical arrow indicated the gradual increase in [Mn(acac)_2_]/[Fe(acac)_3_] from (A)–(G) Mn_3_O_4_ (PDF card no. 01-080-0382).

XRD of Fe_3_O_4_ NPs and Mn_*x*_Fe_3−*x*_O_4_ NPs, which were prepared at a temperature of 200 °C, are shown in Fig. S3.† XRD of the undoped Fe_3_O_4_ NPs, which were formed at 250 °C with an ageing time of 6 h, are shown in Table S1.† The main peaks at the diffractogram of these NPs appear at 21.5° (111), 35.1° (220), 41.4° (311), 50.4° (400), 62.8° (422), 67.3° (511), and 74.1° (440). These peak locations matched ICDD PDF card no. 01-086-2344, revealing the formation of iron oxide (FeO–Fe_2_O_3_).^46–48^ Incorporating Mn ions into the Fe_3_O_4_ lattices as substitutional atoms was then implemented with both Mn(acac)_2_, and Mn(acac)_3_ precursors. By increasing the ratio of [Mn(acac)_2 or 3_] to [Fe(acac)_3_], a small shift of the peaks towards a lower 2*θ* value (closer to the reference peak of MnFe_2_O_4_) was observed in the XRD patterns (Fig. 2 and S4†). Using Mn(acac)_3_ (Fig. S4†) caused a relocation of XRD peak positions closer to the reference peak positions of MnFe_2_O_4_ (2*θ* = 40.8°). This can be attributed to a further inclusion of Mn^3+^ into the spinel iron oxide lattice due to the similar ionic radii between Mn^3+^ and Fe^3+^ (0.64 Å for both (ref. 49)), which are smaller than that of Mn^2+^ (0.80 Å).^49^

With an increase in the Mn precursor concentration, the slight broadening of full-width half maximum (FWHM) of the most intense XRD peaks (311), was observed, which implies a reduction in crystal size.^50^ Calculated size (in diameters) is summarized in Table S1.†

The use of 0.33 ≤ [Mn(acac)_2 or 3_]/[Fe(acac)_3_] ≤ 0.6 resulted in the formation of MnFe_2_O_4_ with an fcc structure as verified by XRD patterns and presented in Fig. 2B–D and S4A–C.† The XRD peaks appearing matched with ICDD PDF card no. 00-010-0319 of MnFe_2_O_4_. The lattice planes correspond to the cubic spinel structure of MnFe_2_O_4_ (ICDD card no. 00-010-0319). For NPs prepared using 1 ≤ [Mn(acac)_2_]/[Fe(acac)_3_] < 7 in Fig. 2E–G, the distinct additional peaks at 2*θ* values of 36.8, 36.9° and 37.0°, respectively, indicated the formation of a secondary phase that was indexed to the (104) Miller plane of MnCO_3_ (ICDD card no. 00-044-1472). In Fig. 2F and G, peaks attributed to the tetraethyleneglycol (TEG) molecule appeared at 2*θ* equal to 27.8° and 27.6° respectively, as shown in the XRD of TEG compound alone before and after thermal treatment (Fig. S5†) as reported by Vamvakidis *et al.*^49^ as well as Khanna and Verma.^51^ Peaks were noticed at 30.2°, 33.1° which were assigned to MnOOH (ICDD PDF card no. 01-074-1631, data are not shown), and MnO_2_ (ICDD PDF card no. 00-024-0735, data are not shown) correspondingly. The presence of multiple phases of Mn oxides/hydroxides was attributed to the formation of H_2_O and Mn_2_O_3_ (the products of thermal decomposition of Mn(acac)_2_),^52^ which can lead to oxidation of Mn^3+^ into Mn^4+^ and hydroxylation of Mn^3+^ oxides.

The increase in the [Mn(acac)_2 or 3_]/[Fe(acac)_3_] led to a slight broadening in the 311 peaks, which indicated a possible alteration of the crystal size,^50,53^ as determined by measuring the FWHM and summarized in Table S1.†^42,53^ The calculated crystal size obtained from XRD of samples (6.5–7 and 5–7.5 nm for NPs prepared from divalent and trivalent Mn precursors, respectively, see PDI of crystal size in Fig. S6†) were within the range of the average particle size derived from TEM. Therefore, these NPs were considered to be single crystalline. However, the XRD analysis indicated the presence of MnCO_3_ for NPs synthesised with precursor ratios in the range of 1 ≤ [Mn(acac)_2 or 3_]/[Fe(acac)_3_] ≤ 3, and the crystal sizes determined by XRD were also within the size range observed by TEM.

### 2.3 Characterization of Mn_*x*_Fe_3−*x*_O_4_ NFs

**Fig. 3:**
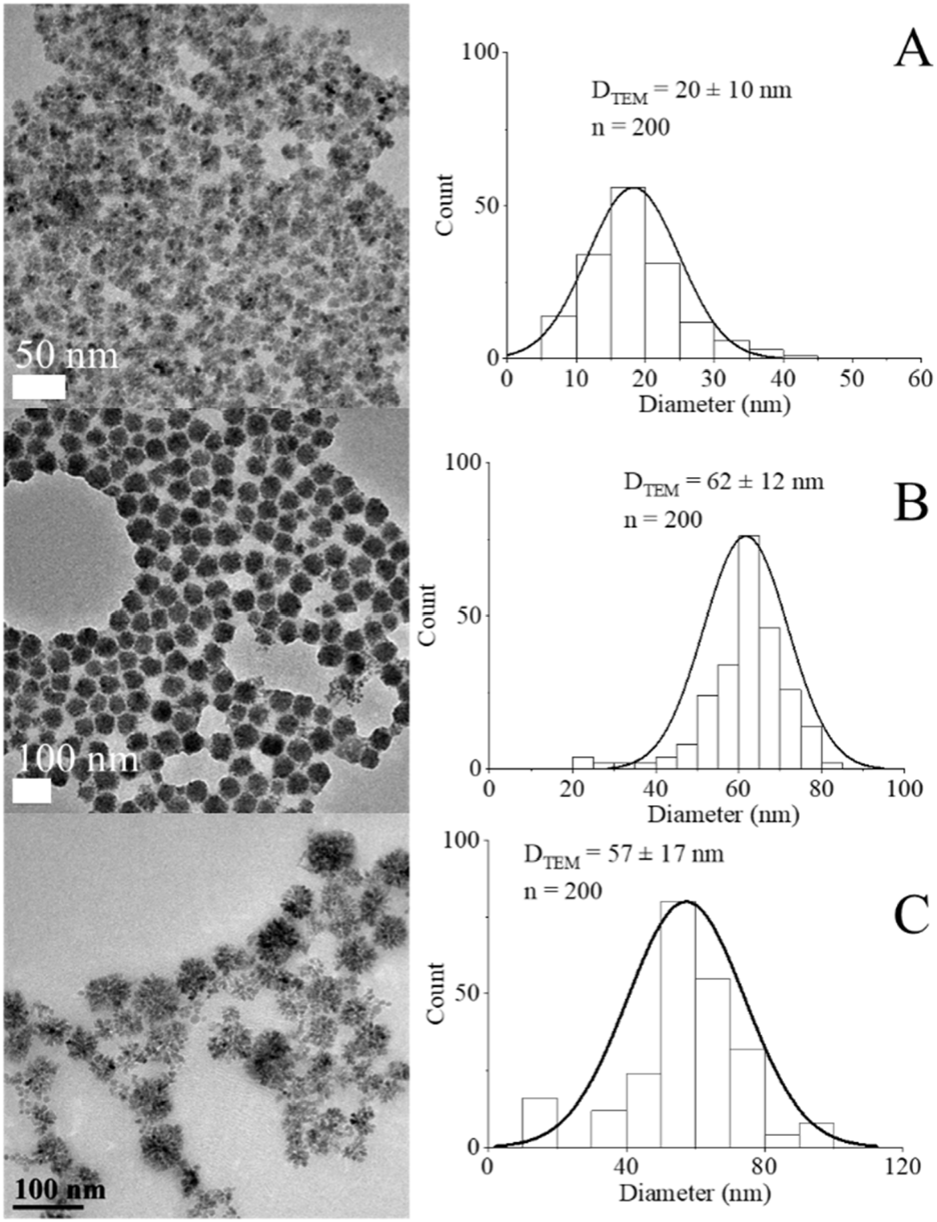
TEM images and histograms for *D*_TEM_ of NFs prepared from [Mn(acac)_3_]/[Fe(acac)_3_] = (A) and (B) 1 & 3, respectively at 200 °C, (C) ratio = 7 at 250 °C.

The preparation of Mn_*x*_Fe_3−*x*_O_4_ NFs was implemented through a modified solvothermal method, and the morphology was precisely regulated by varying the ratios between [Mn(acac)_3_]/[Fe(acac)_3_] precursors as well as the reaction temperature as shown in Fig. 3A and B and [Mn(acac)_2_]/[Fe(acac)_3_] in Fig. S7.†

#### 2.3.1 Morphology of Mn_*x*_Fe_3−*x*_O_4_ NFs

At 200 °C, the polyol solvothermal method resulted in a well-defined flower-like structure with a narrow size distribution, as shown in Fig. 3A and B. The morphology of Mn_*x*_Fe_3−*x*_O_4_ NFs (Fig. 3B) matched the shape of CoFe_2_O_4_ NFs, which was reported by Fu *et al.*, but ours were smaller in size (*D*_TEM_ of M_*x*_Fe_3−*x*_O_4_ NFs = 60 ± 12 nm *vs. D*_TEM_ of CoFe_2_O_4_ NFs = 164.8 ± 20.7 nm).^54^ The smaller diameter of Mn_*x*_Fe_3−*x*_O_4_ NFs than the reported CoFe_2_O_4_ NFs by 2.75 folds can be attributed to sodium hydroxide used during the solvothermal preparation of CoFe_2_O_4_ NFs,^54^ which accelerated the hydrolysis of the precursors and promoted the formation of larger oxide clusters.^55^ TEM analysis of NPs synthesized at 250 °C with a ratio [Mn(acac)_3_]/[Fe(acac)_3_] equal to 7 showed the formation of aggregated crystalline particles in clusters (Fig. 3C).

#### 2.3.2 Crystal structure of Mn_*x*_Fe_3−*x*_O_4_ NFs

**Fig. 4:**
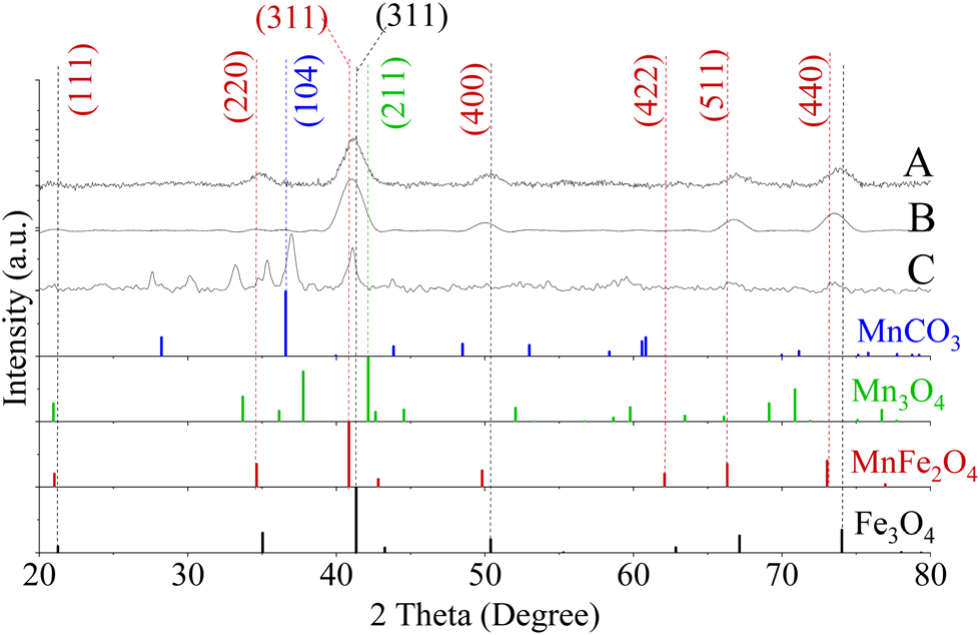
XRD patterns of Mn_*x*_Fe_3−*x*_O_4_ NFs prepared from [Mn(acac)_3_]/[Fe(acac)_3_] ratio equal to (A) 1 and (B) 3 at 200 °C, (C) 7 at 250 °C. A secondary phase matched MnCO_3_ (Reference ICDD PDF card no. 00-044-1472) was found for NFs prepared from a precursor ratio equal to 7 at 250 °C. No detected peaks matched Mn_3_O_4_ (PDF card no. 01-080-0382).

Fig. 4 shows diffraction patterns of Mn_*x*_Fe_3−*x*_O_4_ with crystal size of NFs being in the range 6–8 nm. The small crystal size in comparison to *D*_TEM_ of NFs (Fig. 3) implied the formation of primary nanocrystals, which do not grow significantly. The primary nanocrystals aggregated into larger secondary particles and coarsening, as shown in Fig. 3A–C and as described by Gavilan.^56^ The generation of MnCO_3_ accelerated the hydrolysis of the precursors and promoted the formation of larger oxide clusters.^54^ In our case, nano-clusters were prepared in a single step which included the synthesis of nanoparticles and their coalescence. Shifting in the peak of 311 from the reference Fe_3_O_4_ (PDF card no. 01-089-0688) towards a lower diffraction angle of MnFe_2_O_4_ (PDF card no. 00-010-0319) in response to the increase in [Mn(acac)_3_]/[Fe(acac)_3_] from 1 to 3 was observed, being also an indicator for inclusion of Mn ions into the Fe_3_O_4_ lattices.

In the case of preparing NPs with [Mn(acac)_3_]/[Fe(acac)_3_] ratio equal to 7 at 250 °C, the XRD pattern revealed the formation of a polycrystalline material corresponding to a mixture of phases. As shown in Fig. 4D, the peaks at 36.9° (104) and 27.7° (102) diffraction peaks were indexed to MnCO_3_ (JCPDS card no. 00-044-1472). The 2*θ* Bragg reflections at 21.5° (111), 35.4° (220), 41.0° (311), 50.1° (400), 66.6° (511), and 73.5° (440) confirmed the formation of MnFe_2_O_4_ (JCPDS card no. 00-010-0319). A peak at 27.5° was assigned to TEG51, which was supported by our results, as shown in Fig. S5.† Also, peaks appeared at 2*θ* equal to 30.1°, 33.2° and 35.3° were related to MnOOH (JCPDS card no. 01-074-1631) and MnO_2_ (JCPDS card no. 00-024-0735). Our results revealed that the increase in ratios between the used precursors led to the formation of nano-clusters of Mn_*x*_Fe_3−*x*_O_4,_ which matched what was reported for Mn_*x*_Fe_3−*x*_O_4_ (ref. 57) and other ferrites by a solvothermal method.^58^ The inability of nanocluster formation using [Mn(acac)_2_]/[Fe(acac)_3_] equal to 7 (Fig. S7†) can be attributed to the relative thermal stability of Mn(acac)_2_ which limits its decomposition.^52^

### 2.4 Elemental analysis of Mn_*x*_Fe_3−*x*_O_4_ NPs

**Fig. 5:**
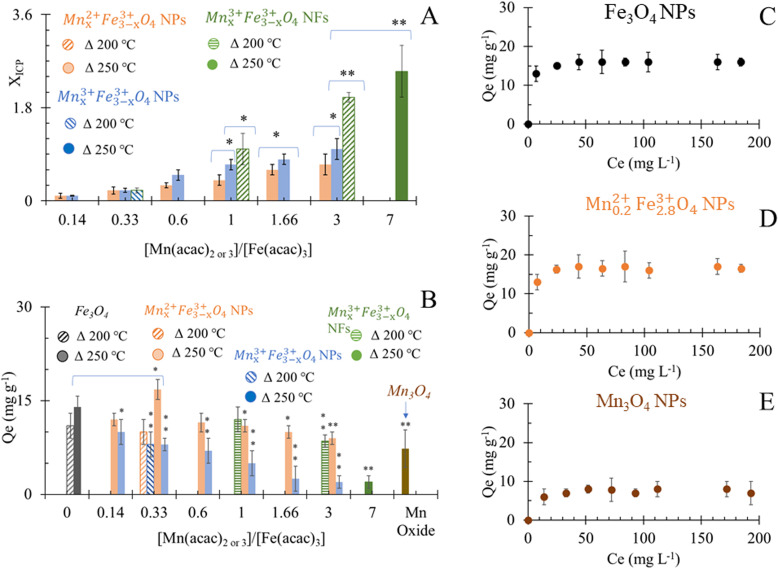
(A) Elemental analysis of Mn_*x*_Fe_3−*x*_O_4_ NS that were prepared at 200 °C and 250 °C. *P < 0.05 and **P < 0.01 in comparison to Mn_*x*_^3+^Fe_3−*x*_^3+^O_4_ NPs of similar precursor ratios; (B) adsorption capacity of NPs for Cr^6+^, *P < 0.05 and **P < 0.01 in comparison to Fe_3_O_4_ NPs; (C)–(E) adsorption isotherm of Cr^6+^ by Fe_3_O_4_, Mn_0.2_^2+^Fe_2.8_^3+^O_4_ and Mn_3_O_4_ NPs respectively.

Results of elemental analyses are presented in Fig. 5A, showing a significant positive relationship between the Mn doping level and [Mn(acac)_2 or 3_]/[Fe(acac)_3_] ratios. The doping level of Mn in Mn_*x*_Fe_3−*x*_O_4_ NPs was probably the reason behind the small shifts in XRD patterns from the reference peak of Fe_3_O_4_ towards a lower diffraction angle of MnFe_2_O_4_ when [Mn(acac)_2 or 3_]/[Fe(acac)_3_] increases as shown in Fig. 2, S3 and S4.† The variation in the Mn doping levels was not significantly affected by the oxidation state of Mn precursor except in the cases when [Mn(acac)_2 or 3_]/[Fe(acac)_3_] ratios were equal to 0.14 and 0.33. The faster thermal decomposition of Mn(acac)_3_ than Mn(acac)_2_ (ref. 52) resulted in more Mn-rich NPs that were prepared by the trivalent Mn precursor than those prepared by the divalent Mn precursor.

Overall, at 250 °C, the change in the oxidation state and the ratios between the precursors did not show a variation in the morphology of NPs, but it significantly affected the Mn doping level. While at a synthesis temperature of 200 °C, the oxidation state and the ratios between the precursors affected both the Mn doping level and resulted in different shapes of Mn_*x*_Fe_3−*x*_O_4_ NPs and NFs.

### 2.5 Functionalisation of NPs and NFs

The advantage of applying a small molecule like citrate as a ligand is that a smaller hydrodynamic radius (*D*_HD_) of NPs can be obtained compared to polymeric ligands, which are reflected in hydrodynamic size (Fig. S8†). Yet, the hydrodynamic shell size of citrate-coated NPs was large enough to maintain a physical barrier leading to good dispersibility. The obtained stable dispersions of nano-colloids were attributed to the negative charges induced by the citrate^59^ as determined by *ζ*-potentials (Fig. S9†).

The most negative value of *ζ*-potential was observed for Mn_*x*_Fe_3−*x*_O_4_ NPs of [Mn(acac)_2 or 3_]/[Fe(acac)_3_] = 3. The colloidal stability was attributed to a weak base (MnCO_3_), as expected from XRD patterns (Fig. 2E–G and 4A) and the negative charge of citrate. For other Mn_*x*_Fe_3−*x*_O_4_ NPs and NFs, the Mn doping level did not show any crucial impact on their *ζ*-potentials.

The FTIR measurements, as shown in Fig. S10,† confirmed that TEG ligand was exchanged by trisodium citrate, similarly observed by Chakraborty *et al.*^60^ Carboxylates exhibited strong absorptions for infra-red spectrum due to their characteristic asymmetric (at 1620–1560 cm^−1^) and symmetric carbonyl stretching (1440 to 1310 cm^−1^). Bands in the region of 1280–1027 cm^−1^ represented the deformation of C–H.^61–63^

### 2.6 Adsorption of Cr^6+^ by NS

The adsorption of Cr^6+^ by Mn*_x_*Fe_3−_*_x_*O_4_ NPs and NFs was studied as a function of the chemical composition and morphology of nanostructures. The quantities of Cr^6+^ (*Q*_e_) adsorbed onto citrate-coated Fe_3_O_4_ NPs were estimated to be 14 ± 1.7 mg g^−1^ at room temperature and pH 7 at equilibrium (Fig. 5B). The smaller size of the NPs presented herein can probably explain the 1.4 fold higher adsorption efficiency compared to that reported by Luther *et al.*^64^ In aqueous solution, Cr^6+^ mainly was present in oxyanion form of chromate (Cr_2_O_7_)^2−^ species and formed sphere complexes with iron oxide *via* surface hydroxyl exchange,^15,36,40,65^ resulting in the generation of monodentate complexes, with simultaneous desorption of surface hydroxyl groups from the metal oxide surface sites.^15,36,40,65^ Upon evaluating commercially available Mn_3_O_4_ as a control material under the same conditions, the binding capacity of Cr^6+^ (7 ± 2.6 mg g^−1^) was significantly lower than to Fe_3_O_4_ which was attributed to the physisorption affinity of Cr^6+^ to such material.^66^

The higher adsorption capacity of NFs in comparison to NPs made of similar [Mn(acac)_3_]/[Fe(acac)_3_] ratio (1 and 3) but at different synthesis temperatures (200 °C *vs.* 250 °C) was attributed to the higher surface area which allows Cr^6+^ to penetrate into NFs.^67^ Increasing the doping level of Mn in NFs causes a decrease in Cr^6+^ adsorption as Mn_0.8_^3+^Fe_2.8_^3+^O_4_ NFs that was prepared from [Mn(acac)_3_]/[Fe(acac)_3_] = 1 can adsorb 12 ± 2 mg g^−1^, while using [Mn(acac)_3_]/[Fe(acac)_3_] = 3 resulted in NFs with adsorption capacity equal to 8.5 ± 2 mg g^−1^.

Under the employed conditions, only Mn_0.2_^2+^Fe_2.8_^3+^O_4_ (*x* = 0.2), prepared at 250 °C was comparable to adsorption capacity to Fe_3_O_4_ NPs (16.8 ± 1.6 *vs.* 14 ± 1.7 mg g^−1^). The use of Mn_0.2_^2+^Fe_2.8_^3+^O_4_ NPs improved the Cr^6+^ adsorption by over 2 fold compared to Mn_3_O_4_, as shown in Fig. 5B. The adsorption of chromate anions was due to the formation of weak bonds with Mn_*x*_Fe_3−*x*_O_4_ substrate.^40^ The adsorption capacity of Cr^6+^ by stoichiometric MnFe_2_O_4_ NPs was reported to be higher than by non-stoichiometric Mn_1−*x*_Co_*x*_Fe_2_O_4_ (*x* = 0.2, 0.4 and 0.6).^68^ Our results are in agreement with what was stated by Martinez-Vargas *et al.*^69^ as non-stoichiometric Mn_0.25_Fe_2.75_O_4_ NPs exhibited the best adsorption capacity to As^3+^. The surface of Mn_0.2_^2+^Fe_2.8_^3+^O_4_ has been reported to be rich in hydroxyl groups^69^ which favour Cr^6+^ adsorption. The colloidal dispersion of our NPs and their small diameter can explain the reason behind their better adsorption capacity compared to other reported MnFe_2_O_4_ NPs (15 mg g^−1^ (ref. 36) and 13.54 mg g^−1^ (ref. 70)), while it is also comparable with results from other reports (18.02 mg g^−1^).^40^ The increase in the Co substitution for iron in magnetite (Fe_3−*x*_Co_*x*_O_4_, 0 ≤ *x* ≤ 1) enhanced the adsorption capacity of NPs to Cr^6+^ slightly.^71^ Increasing the zinc content in magnetite (Fe_3−*x*_Zn_*x*_O_4_, *x* = 0, 0.25, 0.49) has been reported to initially decrease the Cr removal efficiency, but Fe_2.26_Zn_0.74_O_4_ and Fe_2.1_Zn_0.99_O_4_ led to its improvement.^72^

Except Mn_0.2_^2+^Fe_2.8_^3+^O_4_, Mn_*x*_Fe_3−*x*_O_4_ NPs prepared from both Mn sources showed an inverse trend for adsorption capacity of Cr^6+^ with the increase of *x* in comparison to Fe_3_O_4_ NPs. The inverse relationship between Mn concentration in the ferrite composition and adsorption of heavy metals was also observed in the case of arsenic adsorption by Mn_*x*_Fe_3−*x*_O_4_ NPs and was attributed to low binding affinity to the As.^69^ Introducing Mn into ferrite reduced the adsorption capacity of Fe_3_O_4_ to Cr^6+^ from 15.9 mg g^−1^ to 8.54–8.9 mg g^−1^.^64,70^ It was suggested that the release of Mn cations into the solution as a result of reduction of Cr^6+^ (ref. 64) alters the surface structure. The decrease in Mn doping in the Mn_1−*x*_Co_*x*_Fe_2_O_4_ (*x* = 0.2, 0.4 and 0.6) induced a progressive, positive impact on the adsorption efficiency of Cr^6+^. Since Mn^2+^ ions have larger ionic radii than Co^2+^ (0.8 Å *vs.* 0.7 Å), the increase in *x* turned the overcoming of energy barriers for ion exchange interaction more difficult.^68^ Given that the physical mechanism of Cr^6+^ adsorption on the surface of oxide was reported to be a combination of electrostatic interactions between charged oxides and Cr^6+^ and ion exchange in the aqueous solution,^40^ the increase in Mn doping level showed a negative impact on Cr^6+^ adsorption by Mn_1−*x*_Co_*x*_Fe_2_O_4_.^68^ At higher dopant concentration *x* = 0.8, more CoFe_2_O_4_ was proposed to be formed on the surface.^68^ Considering the larger ionic radii of Mn^2+^ cation than Fe^3+^ (0.80 Å *vs.*^49^ 0.64 (ref. 49)), our results can be explained on the basis of the reverse impact of Mn doping level on the adsorption capacity of Mn_*x*_Fe_3−*x*_O_4_ NPs to Cr^6+^. Yet, in the case of Mn_*x*_^3+^Fe_3−*x*_^3+^O_4_, the ionic radius of Mn^3+^ is smaller than Mn^2+^ and approximately equal to Fe^3+^ radius but Mn_*x*_^3+^Fe_3−*x*_^3+^O_4_ NPs showed lower adsorption capacities than their Mn_0.2_^2+^Fe_2.8_^3+^O_4_ NPs counterparts. The inferiority of Mn^3+^ in the adsorption of Cr^6+^ could be attributed to the lower redox potential of Mn^3+^/Mn^2+^ (+1.5 V) than Fe^3+^/Fe^2+^ (+1.9 V).^49^ Mn^2+^ has half-filled 3d orbital ([Ar] 3d^5^ 4S^0^), which makes it more stable than Mn^3+^ ([Ar] 3d^4^ 4S^0^). While, oxidizing Fe^2+^ ([Ar] 3d^6^ 4S^0^) of Fe_3_O_4_ NPs into more stable Fe^3+^ ([Ar] 3d^5^ 4S^0^) is favored and can support the possible redox-based adsorption of Cr^6+^.

The adsorption of Cr^6+^ by the selected citrate-coated adsorbents that showed the best *Q*_e_ at pH 7 at room temperature can be described by Langmuir isotherm model as a function of the initial Cr^6+^ concentrations (Fig. 5C–E). Hence, the surface of nano-sorbents has homogeneous energy distribution *via* a monolayer sorption process. The calculated maximum adsorption capacity (*Q*_max_) by Langmuir isotherm model fitted the results of *Q*_e_.

### 2.7 The Raman spectrum of Mn_0.2_^2+^Fe_2.8_^3+^O_4_ NPs

Raman spectroscopy is also a useful tool that provides further structural details.^73^ Raman spectra of representative Fe_3_O_4_ and Mn_0.2_^2+^Fe_2.8_^3+^O_4_ NPs were recorded. The Raman spectrum of Fe_3_O_4_ (Fig. S11A†) expressed 5 Raman active modes including 190 (T_2g_(1)), 340 (E_g_), 490 (T_2g_(2)), 540 (T_2g_(3)) and 670 cm^−1^ (A_1g_). For Mn_0.2_^2+^Fe_2.8_^3+^O_4_ NPs, the broad A_1g_ band involved two modes centered at 595 and 670 cm^−1^ due to the presence of Mn and Fe cations. The Raman shift at 220 cm^−1^ in Fig. S11B† showed an induced phase transition at the surface of the Mn_0.2_^2+^Fe_2.8_^3+^O_4_ NPs due to the laser's power.^74^

### 2.8 The oxidation state of Mn and Fe in Mn_0.2_^2+^Fe_2.8_^3+^O_4_ and Mn_*x*_^3+^Fe_3−*x*_^3+^O_4_ NPs

XPS was utilized to gain insights into the chemical composition and oxidation state of the selected Mn_*x*_Fe_3−*x*_O_4_ NPs, which have either maximum or minimum Cr^6+^ adsorption capacity (Fig. 5). Binding energies (BE) were used to identify different elements and their valence states. In Fig. S12,† the wide-scan spectra of Mn_0.2_^2+^Fe_2.8_^3+^O_4_ and Mn_*x*_^3+^Fe_3−*x*_^3+^O_4_ NPs indicate the presence of carbon (C) and oxygen (O) elements besides Mn and Fe. Using the relative area under the deconvoluted XPS bands, a semi-quantitative estimation of the valence states of the elements in the mixed-valence compounds was achieved.

The presence of C was identified by BEs of C 1s around 284.6, characteristic energies correspond to C–C, C–O–C, O–C=O, and C=O bonds which are due to the presence of surfactant. The presence of O in the XPS spectra was attributed to the metal oxide itself, hydroxyl bonded to metal or adsorbed H_2_O as was expressed by BE of O 1s at 529.7 eV. Other BE appeared at 531.0, 532.3, and 535.3 eV were ascribed to C=O and C–O bonds coming from the ligand.

#### 2.8.1 The oxidation state of Mn

**Fig. 6:**
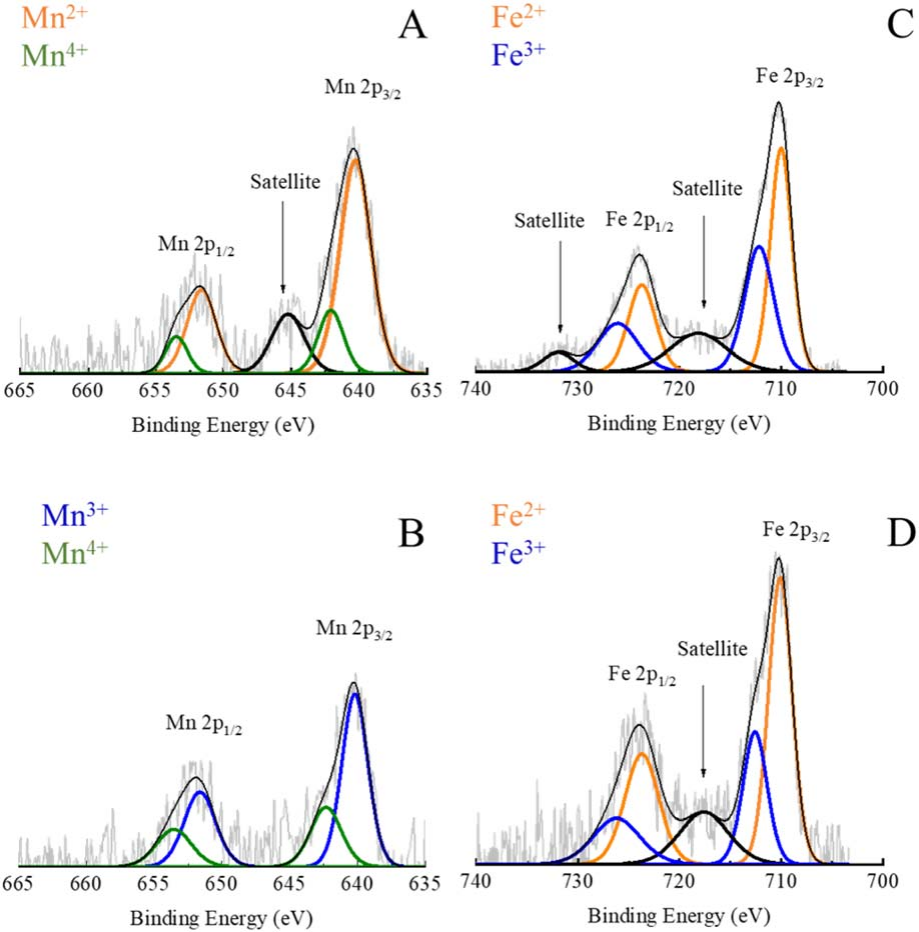
High-resolution XPS spectra of Mn 2p in Mn_0.2_^2+^Fe_2.8_^3+^O_4_ (A); Mn 2p in Mn*_x_*^3+^Fe_3−_*_x_*^3+^O_4_ NPs (B); Fe 2p in Mn_0.2_^2+^Fe_2.8_^3+^O_4_ (C) and Fe 2p in Mn*_x_*^3+^Fe_3−_*_x_*^3+^O_4_ NPs (D).

In Fig. 6A, Mn 2p was fitted by 5 contributions at around 640.2, 642.1, 645.2, 651.6 and 653.4 eV. The broadening of peaks demonstrated that Mn is present in an oxide form rather than a metallic one.^75,76^ The asymmetric Mn 2p_3/2_ main metal peak at 640.2 eV was subjected to a 2p_3/2_ to 2p_1/2_ splitting of FWHM 2.7 and 2.1 eV, respectively. Binding energies of 640.2 and 651.1 eV were reported to be related to Mn 2p_3/2_ and Mn 2p_1/2_ of Mn^2+^ correspondingly.^40,75–77^ The small satellite peak at 645.2 eV (≈14%, 2.9 eV FWHM, Table S2†) was assigned to MnO. Since stoichiometric Mn_*x*_Fe_3−*x*_O_4_ can be expressed as MnO–Fe_2_O_3_, this points to the formation of Mn_*x*_Fe_3−*x*_O_4_ NPs^75^ in agreement with XRD results (Fig. 2B).

Nevertheless, in the case of Mn_*x*_^3+^Fe_3−*x*_^3+^O_4_ NPs, the BE of Mn 2p was fitted by 4 peaks which are located at 640.2, 642.1, 651.6 and 653.4 eV, as shown in Fig. 6B. The absence of a small satellite peak at 645.2 was reported due to the formation of either Mn^3+^ or Mn^4+^.^30^

A partial oxidation of Mn^2+^ to Mn^3+^ has been reported for Mn_*x*_Fe_3−*x*_O_4_ NPs prepared *via* polyol solvothermal method using Mn(acac)_2_ precursor^42^ due to the oxidative atmosphere inside the autoclave.^49^ Both divalent and trivalent Mn cations were reported to be present in Mn_*x*_Fe_3−*x*_O_4_ NPs with a strong preference for tetrahedral sites and octahedral positions for Mn^2+^ and Mn^3+^, respectively.^42^ Hence, trivalent Mn was supposed to be formed during the synthesis of NPs as suggested by XRD and matched MnCO_3_ ICDD PDF card no. 00-010-0319. The relatively lower amount of Mn^2+^ in Mn^3+^-substituted ferrites could account for the fact that Mn_*x*_^3+^Fe_3−*x*_^3+^O_4_ NPs showed lower Cr^6+^ adsorption capacity than Mn^2+^-substituted ferrites (Fig. 5B).

#### 2.8.2 The oxidation state of Fe

For both samples, as shown in Fig. 6C and D, a peak of Fe 2p_3/2_ for Fe^3+^ was spotted at 710 eV, and its satellite appeared at 718 eV. The asymmetric peaks are situated at 723.6 eV, attributed to Fe^3+^ 2p_1/2_. For Fe 2p_1/2_, another satellite peak was observed at 729.5 eV. The peak of Fe 2p_1/2_ was wider and weaker than Fe 2p_3/2_ peak, and the FWHM of Fe 2p_1/2_ peak is smaller than that of Fe 2p_3/2_ because of spin–orbit (*j*–*j*) coupling. From the calculated FWHM (Table S3†), the FWHM of Fe 2p peaks at 712 eV was slightly smaller than its counterpart at 712 eV, which can serve as an indicator for the presence of both Fe^2+^ and Fe^3+^ in these two samples.^76,78^ This interpretation matched the elemental analysis results by ICP (Fig. 5A) for the formation of non-stoichiometric Mn_*x*_Fe_3−*x*_O_4_. Furthermore, the absence of the satellite peak at 732 eV, as shown in Fig. 6D, was attributed to the presence of Fe_3_O_4_.^78^

Mn_0.2_^2+^Fe_2.8_^3+^O_4_ NPs adsorb Cr^6+^*via* an ion exchange between the hydroxyl groups on the surface of NPs and chromate (Cr_2_O_7_)^2−^ oxyanion.^15,36,40,65^ The adsorption of Cr^6+^ on iron oxides/hydroxides was reported to generate inner-sphere coordination complexes,^79,80^ in which chromates are linked to a central metal atoms (or ions) by covalent bonds. Fe forms monodentate (one covalent bond) and bidentate (two covalent bonds) complexes with chromates.^79,80^ The inner-sphere complex is strong and non-reversible.^79^ The reduction of adsorbed Cr^6+^ to Cr^3+^ by Fe^2+^ or Mn^2+^ resulted in the formation of precipitated Cr(OH)_3_ or Cr_*x*_Fe_1−*x*_(OH)_3_.^72^ So, there is a possibility of the presence of Cr^6+^ and Cr^3+^ on the surface of NS.

Due to the highest adsorption capacity of Mn_0.2_^2+^Fe_2.8_^3+^O_4_ NPs, this sample was selected to explore its enhancement impact on the bio-reduction of Cr^6+^ by *S. oneidensis* MR-1 in comparison to undoped Fe_3_O_4_ and Mn_3_O_4_ NPs.

### 2.9 The lethal dose of Cr^6+^ for the tested *Shewanella*

*Shewanella* bacterial species are considered metal-reducing and resistant bacteria.^26^ In our work, results revealed that the minimum inhibition concentration (MIC) of Cr^6+^ for the tested wild-type *Shewanella* (*S. oneidensis* and *S. loihica* PV-4, see the molecular identification (Table S4†)) was 60 mg L^−1^ and 70 mg L^−1^ respectively, being slightly higher than what was reported previously.^26^ For *S. oneidensis* JG1486 and JG3355 (molecular identification at ESI†), MICs were 20 mg L^−1^ and 5 mg L^−1^, respectively. The bactericidal effect of Cr^6+^ was documented because of being taken up by *Shewanella* intracellularly and caused cell lysis. In fact, the toxic effect of Cr^3+^ appeared to be associated with extracellular interactions, leading to stress-associated cell morphology and then to a lethal effect.^26,81–83^ Before reacting with Cr^6+^, the wild-type *S. oneidensis* MR-1 and *S. loihica* PV-4 cells were reported to be regular small rod-shaped with smooth surfaces.^26,81–83^ Meanwhile, the bacterial cells changed to be atrophic with a shrunken-surface shape and crack formation was also observed after the reaction.^26,81,83^ However, *S. loihica* PV-4 cells were reported to be elongated and exhibited a rough surface upon exposure to Cr^6+^,^82^ which can explain why *S. loihica* PV-demonstrated higher resistance and reduction ability for Cr^6+^. As hazardous metal ions could damage microbial DNA when they entered the cells, extracellular reduction benefitted *Shewanella* for their survival.^82^ The low resistance of mutants was due to the inability of bio-reduction of Cr^6+^ for JG1486,^84^ but the possibility of the presence of other stress regulators made such mutants more resistant regarding JG3355.^85,86^

### 2.10 Effect of the selected NPs on the viability of *Shewanella* in response to the sublethal concentration of Cr^6+^

**Fig. 7:**
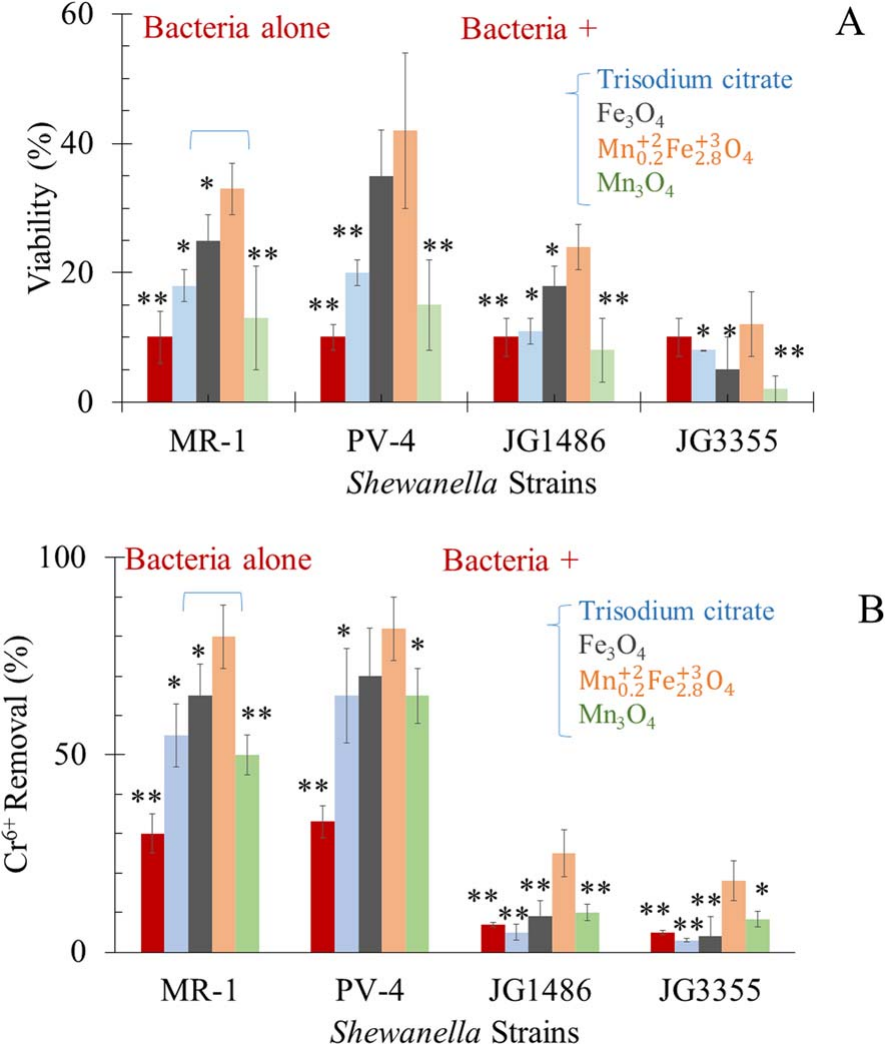
In the presence of the tested agents (A) viability of tested *Shewanella* under sublethal dose of Cr^6+^ (B) removal of Cr^6+^ by *Shewanella* strains. *P < 0.05 and **P < 0.01 in relation to the impact of Mn_0.2_^2+^Fe_2.8_^3+^O_4_ NPs in each data set separately.

In the absence of Cr^6+^, tri-sodium citrate alone, citrate-coated Mn_0.2_^2+^Fe_2.8_^3+^O_4_, Fe_3_O_4_ and Mn_3_O_4_ can sustain the viability of bacteria. At a sub-lethal concentration of Cr^6+^, the viability of wild-type bacteria was improved in the tested groups amended by citrate alone (only 10–12%), as illustrated in Fig. 7A*.* Bencheikh-Latmani *et al.*^87^ explained a similar observation as a result of the complexation between the product of bio-reduction (Cr^3+^) and citrate, which consequently limits the availability of the toxic metal to bacterial cells.^87^ In response to Cr^6+^ toxicity, Mn_0.2_Fe_2.8_O_4_, Fe_3_O_4_ and Mn_3_O_4_ NPs improved the viability of *S. oneidensis* JG1486 strain by 3.3, 2.5, 1.3 folds, and of *S. oneidensis* JG5533 strain by 1.2, 0.5, 0.2 folds, respectively.

### 2.11 Bio-reduction of Cr^6+^ by tested *Shewanella*

For safe removal of Cr^6+^, such hexavalent cations should be detoxified by reduction to Cr^3+^. The capability of *S. oneidensis*^83^ to respire Cr^6+^ was affected by the initial concentration of the heavy metal.^83,88,89^ These results were credited for the chromate dose-dependent toxicity, which causes growth and viability inhibition.^83,88^ This occurred in the presence of Cr^6+^ alone^83,88,89^ or in the presence of goethite and humic acid^34^ or ferric oxyhydroxide mediators.^83,88^ Hence, our experiments were designed at a high concentration of Cr^6+^, *i.e.* sub-lethal dose.

Our results (Fig. 7B) revealed a significant drop in the concentration of Cr^6+^ in media supplemented by both wild-type of *Shewanella* which included the strain of interest (*S. oneidensis* MR-1) and positive control (*S. loihica* PV-4); this was attributed to the respiration of Cr^6+^ into Cr^3+^ form^81,90,91^ or bio-sorption^92,93^ by bacterial cells. The drop in the concentration of Cr^6+^ in the medium exposed to *S. oneidensis* JG1486 and JG3355 was significantly lower than those supplemented by both wild type bacteria.

The tested *Shewanella* oxidized lactate (electron source), and the liberated electrons are transferred *via* the respiratory chain to be directed to an externally available terminal electron acceptor (Cr^6+^). The redox potential of Cr^6+^ (1.33 V *vs.* standard hydrogen electrode; SHE) has been reported to be higher than the redox potential of oxygen (1.23 V) and the electron source (−0.19 V).^94^ So, Cr^6+^ was considered a favourable electron acceptor for bacteria in the process of respiration as bacteria gain more energy.^94^

Cr^6+^ can be reduced extracellularly^82^ and also transported into the cell interior and then reduced in the cytoplasm.^82^ The ability of *Shewanella* to transfer electrons to metal ions was known to take place *via* one of four porin–cytochrome conduits; the MtrCAB complex,^95^ the MtrFED complex,^96^ the DmsEFA dimethyl sulfoxide reductase system^97^ and the SO4359–SO4360 system.^96^ The superiority of *S. loihica* PV-4 in the respiration of Cr^6+^ in our experiment was thanks to their higher content of c-type cytochrome genes in the metal reductase-containing locus than *S. oneidensis* MR-1.^98^*S. oneidensis* JG1486 (ΔmtrCAB/ΔmtrFED/ΔomcA/ΔdmsE/ΔSO4360/ΔcctA/ΔrecA) lacks the responsible genes for extracellular metal reduction.^84^ The recombination between the expression of outer membrane cytochromes (controlled by lac promoters) and periplasmic electron carriers was stopped by the deletion of *RecA* gene.^84^ Such mutants showed the lowest removal of Cr^6+^ as a result of the inability of bioreduction.^84^*S. oneidensis* JG3355 lacked both ClpX and ClpP genes.^86^ The role of ClpXP has been revealed for regulating Fe^2+^ stress in anaerobic bacteria^86^ and stress regulation (ClpP) in response to 24 h Cr^6+^ exposure.^85^ Therefore, the inability of *S. oneidensis* JG3355 to respire metal could be the result of losing the bacterial viability as was reported before^29–31^ and presented in Fig. 7A.

### 2.12 Enhancement of respiration of Cr^6+^ by *Shewanella* in the presence of selected materials

The respiration of Cr^6+^ (at sub-lethal concentration) was improved in the tested groups supplied by citrate alone (only 0.83 folds), as illustrated in Fig. 7B. Bencheikh-Latmani *et al.*^87^ explained a similar observation as a result of the possible complexation between the product of bio-reduction (Cr^3+^) and citrate, which consequently limits the availability of the toxic metal to bacterial cells.^87^

In the presence of a sublethal concentration of Cr^6+^, the alive cell extent was the highest in the group of citrate coated Mn_0.2_^2+^Fe_2.8_^3+^O_4_, Fe_3_O_4_ and Mn_3_O_4_ (in descending order). The presence of Mn_0.2_^2+^Fe_2.8_^3+^O_4_ as adsorbent was beneficial to microbial survival, which was positively related to enhanced Cr^6+^ bio-reduction by 2.5–3.6 folds. The increase in the percentage of bacterial viability may be attributed to the adsorption of Cr^6+^ by NPs, which led to the decrease of stress on the strains themselves. In addition, the possible continuous adsorption–desorption rate of Cr^6+^ was based on Langmuir adsorption isotherm of the equilibrium between the adsorbate and adsorbent system (Fig. 5C–E).

The presence of manganese in the chemical structure of NPs improved the antioxidant activity and, in turn, the viability of cells and the ability to respire metal.^99^ Mn^2+^ ions can act as antioxidants which helps enzymatic systems to act against oxidative stress. For Fe-rich and Mn-poor cells such as *S. oneidensis* MR-1, death at low doses of ionizing radiation might not be caused by DNA damage inflicted during irradiation but instead by the release of Fe^2+^ and the subsequently formed toxic by-product of energy-metabolism after irradiation.^100^

The electron transfer from cells to the acceptor^101^ occurred *via* redox cycling of the electron-donating and accepting functional groups *via* direct electron transfer through NPs. The affinity of MnFe_2_O_4_ NPs to bind proteins on the bacterial outer membrane can improve the contact area between a single bacterium cell and an external electron acceptor.^37^ There are some explanations for NP-enhanced bio-reduction of Cr^6+^ to Cr^3+^by *S. oneidensis* MR-1; however, the exact mechanism is not fully unravelled.^102^ NPs can act as a bridge between the bacterial cell and Cr^6+^ to promote electron transfer.^102^ Mn_0.2_^2+^Fe_2.8_^3+^O_4_ NPs can adsorb Cr^6+^*via* ion exchange^40,103^ and covalent bonding of Cr^6+^ on their surfaces.^36^ The adsorption of Cr^6+^ on the surface of NPs and its reduction to Cr^3+^ decreases the availability and toxicity of Cr^6+^, which improves the efficiency of microbial respiration.^90,104^ Since MnFe_2_O_4_ NPs have electrochemical properties,^37,38^ they can link *S. oneidensis* MR-1 with Cr^6+^ as an electron mediator from the cell to Cr^6+^, a terminal electron acceptor. In MnFe_2_O_4_, the existence of Mn and Fe in different oxidation states facilitates the redox processes on the NP surface.^103^ Finally, *S. oneidensis* MR-1 can reduce Fe^3+^of Mn_0.2_^2+^Fe_2.8_^3+^O_4_ NPs to Fe^2+^, which can further reduce Cr^6+^ to Cr^3+^.^90^ The bio-genic Fe^2+^ can reduce Cr^6+^ leading to releasing Fe^3+^ into the medium and the dissolution of NPs.^88^

## 3. Experimental section

### 3.1 Materials

All chemicals were used as received without further purification.

#### 3.1.1 For nanoparticle synthesis

Hydrochloric acid (HCl, 34%), and acetone (C_3_H_6_O, >99%), were bought from VWR Chemicals, UK. Anhydrous sodium hydroxide (NaOH, 98%), iron(iii) acetylacetonate (Fe(acac)_3_, 99.9%), manganese(ii) acetylacetonate (Mn(acac)_2_, 99.9%), manganese(iii) acetylacetonate (Mn(acac)_3_, 99.9%), tetraethylene glycol (TEG, 99%), manganese(ii,iii) oxide (Mn_3_O_4_, 97%), manganese standard for inductively coupled plasma (ICP), TraceCERT®, 1000 mg L^−1^ Mn in HNO_3_, iron standard for ICP (TraceCERT®, 1000 mg L^−1^ Fe in HNO_3_), iron chloride tetrahydrate (FeCl_2_·4H_2_O ≥ 99%), nitric acid (HNO_3_, 70%), potassium bromide (KBr, FTIR grade 99%), tri-sodium citrate dihydrate (Na_3_C_6_H_5_O_7_·2H_2_O), hydroxylamine hydrochloride (ACS reagent, 98.0%), sodium acetate (Na-acetate, anhydrous, ReagentPlus®, ≥99.0%) and 1,10-phenanthroline monohydrate (titration ≥ 99.5%) were obtained from Sigma-Aldrich (UK). Absolute ethanol (C_2_H_5_ OH, 99.9%) was purchased from HaymanKimia, UK.

#### 3.1.2 For Cr^6+^ adsorption

1,5-Diphenylcarbazide (DPC, ≥98.0%) was obtained from Sigma-Aldrich (UK), and potassium dichromate (K_2_CrO_4_) was purchased from VWR Chemicals, UK.

#### 3.1.3 For microbiological studies

Both forward primer (1369F, CGGTGAATACGTTCYCGG) and reverse primer (1492R, GGWTACCTTGTTACGACTT) were obtained from Integrated DNA Technology (UK) in dry forms. Vitamin Supplement (ATCC® MD-VS™) and trace Mineral Supplement (ATCC® MD-TMS™) were procured from American Type Culture Collection (ATCC, UK). M9 minimal salts (2×) medium, Invitrogen Qubit assay kits, LIVE/DEAD BacLight Bacterial viability assay, and Invitrogen ultrapure DNase/RNase-free sterile distilled water were purchased from Thermo Fisher, UK. Luria–Bertani agar (LB) medium was supplied from Oxoid, UK. 4-(2-Hydroxyethyl)-1-piperazine-ethanesulfonic acid (HEPES, ≥99.5%), sodium dl-lactate (Na-lactate, ≥99.0%), and sodium fumarate dibasic (Na-fumarate, ≥99.0%) were obtained from Sigma-Aldrich (UK). FastDNA Spin Kit for Soil was purchased from MP Biomedicals, UK, and Luna Universal qPCR Master Mix was obtained from New England Biolabs, UK. Microbank™ cryobeads was purchased from Pro-Lab Diagnostics, UK.

#### 3.1.4 Sources of bacteria of interest

Freeze-dried cultures of *Shewanella oneidensis* MR-1 (strain number LMG 19005) were purchased from Belgian Coordinated Collections of Microorganisms/Laboratory for Microbiology of the Ghent University (BCCM/LMG). *Shewanella loihica* PV-4 (strain number DSMZ 17748) was obtained from Leibniz-Institut DSMZ-Deutsche Sammlung von Mikroorganismen und Zellkulturen GmbH and used as a positive biological control for microbial reduction of Cr^6+^. *S. oneidensis* JG1486 and JG3355 were kindly provided as LB agar stabs cryovials by Professor Jeffrey A. Gralnick at University of Minnesota, USA and used as negative biological controls. The dried bacteria were recovered *via* streaking on LB agar plate and incubated at 37 °C for 18 h. Colonial growth from all cultures was stored after being preserved on cryobeads at −20 °C.

### 3.2 Synthetic methodology

Mn_*x*_Fe_3−*x*_O_4_ nanostructures (NS) were prepared by a polyol solvothermal synthetic procedure^42,49^ with some modifications. The impact of the oxidation state of Mn precursors, *i.e.* Mn(acac)_2_ and Mn(acac)_3_, the molar ratio between [Mn precursor] to [Fe(acac)_3_] and reaction temperature on the nanoparticle properties were studied. Based on previous experience from our research group, using 15 wt%/vol as a total dissolved precursor concentration resulted in NPs with narrow size distribution.^46^

#### 3.2.1 Synthesis of Mn_*x*_Fe_3−*x*_O_4_ NPs

The desired amounts of precursors with ratios [Mn(acac)_2 or 3_]/[Fe(acac)_3_] equal to 0, 0.14, 0.33, 0.6, 1, 1.66 and 3 were mixed in 20 mL of TEG as a solvent*.* The resulting mixture was processed by vortexing for 10 min, then sonicated for 30 min to be homogenized, followed by its transfer into a 45 mL Teflon-lined stainless-steel autoclave. The autoclave was placed in an oven (Memmert, model UFP400) at room temperature, and the reaction temperature was raised for 30 min to 250 °C, which was maintained for 6 h. For the ratio [Mn(acac)_2 or 3_]/[Fe(acac)_2_] equal to 0 and 0.33, the temperature was raised up to 200 °C only. The resulting black dispersion was separated by a magnet and washed with 1 : 10 v/v of acetone, followed by ethanol and water three times for each solvent. Then, the nanomaterials were ready for characterization and functionalization.

#### 3.2.2 Synthesis of Mn_*x*_Fe_3−*x*_O_4_ NFs

Mn_*x*_Fe_3−*x*_O_4_ NFs were prepared following the above-described protocol for Mn_*x*_Fe_3−*x*_O_4_ NPs and literatures^54^ with some modifications. The autoclave was inserted in the oven, which was heated up to 200 °C for 6 h, and the tested ratios between precursors [Mn(acac)_3_]/[Fe(acac)_3_] were 1 and 3 while [Mn(acac)_2 or 3_]/[Fe(acac)_3_] ratio of 7 was kept at 250 °C for 6 h.

#### 3.2.3 Functionalization of Mn_*x*_Fe_3−*x*_O_4_ NS

In order to exchange the initial ligand TEG, 1 mL of the dispersions of the prepared nanomaterials and 10 mL of 1 M aqueous tri-sodium citrate solution was mixed for 48 h at room temperature under stirring. Immobilization of citrate on the surface of the commercially available Mn_3_O_4_ (used as Mn-rich and iron-free ferrite control) was carried out by dispersing 0.1 g of the metal oxide in 10 mL of 1 M aqueous tri-sodium citrate solution under similar mentioned conditions. The functionalized nanostructures were purified by magnetic separation, followed by washing with acetone three times, after that ethanol washing was performed three times, and finally the particles were dispersed in de-ionized water.

#### 3.2.4 Characterization of Mn_*x*_Fe_3−*x*_O_4_ NS

For the prepared nanomaterials, the shape and diameter of the core were determined by a JEOL JEM 1200-EX microscope operating at an acceleration voltage of 120 kV. The crystal phase and the average crystallite size were analyzed by X-ray diffractometer (XRD; PANalytical XPERT PRO MPD) coupled with Co Kα radiation source (*λ* = 1.789 Å) and an X'Celerator detector operated at 40 kV and 40 mA. The crystalline phases were identified using the International Centre for Diffraction Data Powder Diffraction File (ICDD PDF) database. The crystal domain size (*D*_XRD_) was calculated using Scherrer's equation at the most intense X-ray peaks (311). The chemical composition of Mn_*x*_Fe_3−*x*_O_4_ was determined by an Optima 3100 XL PerkinElmer Inductively Coupled Plasma Atomic Emission (ICP-AES) spectrometer. The oxidation states of Mn and Fe in selected Mn_*x*_Fe_3−*x*_O_4_ NPs that produce the lowest and the highest *Q*_e_ were analysed by X-ray photoelectron spectrometry (XPS), a Kratos Analytical AXIS Ultra DLD system with aluminium monochromatic X-ray source (Al_Kα_ = 1486.6 eV), under ultra-high vacuum conditions (10^−9^ Torr). The experimental curves were best fitted by a combination of Gaussian (70%) and Lorentzian (30%) distributions. Over the range 150–2000 cm^−1^, Raman spectra were collected for powder samples of the selected Mn_*x*_Fe_3−*x*_O_4_ materials prepared using [Mn(acac)_2_]/[Fe(acac)_3_] equal to 0 and 0.33 at 250 °C for 6 h. A Renishaw InVia micro-Raman spectrometer was used and experiments were conducted at room temperature and excited by green Ar-laser for excitation (*λ* = 514.5 nm) of photon energy 2.4 eV and diffraction grating 2400 grating.

#### 3.2.5 Surface characterization of Mn_*x*_Fe_3−*x*_O_4_ NS

In order to study the surface coordination of the capping agents, a PerkinElmer FTIR; Spectrum 100 instrument with a Ge/Ge universal attenuated total reflectance (ATR) was used. The samples were prepared by air drying at room temperature overnight to yield a fine powder and then directly placed on an ATR crystal. The measurement window for the recorded spectra was in the range 4000–600 cm^−1^, with a 2 cm^−1^ resolution, using 40 scan accumulation. The hydrodynamic diameter (*D*_HD_) of citrate-functionalized NPs was evaluated by DLS measurements performed with a Nanosizer ZS instrument (He–Ne 633 nm laser) from Malvern Instruments Ltd, Worcestershire, UK). The *ζ*-potentials of the functionalized NPs were determined using a disposable capillary cell (DTS1070) at 25 °C by DLS. For iron content quantification of the functionalized nanomaterials dispersed in water, a colourimetric phenanthroline method was applied for the acid-digested tested agent.^105^ The concentration of Mn in the Mn_3_O_4_ dispersion was estimated by inductively coupled plasma atomic emission spectroscopy (ICP-AES) spectrometer.

#### 3.2.6 Measurement of the Cr^6+^ adsorption capacity of nanostructures

Equal volumes of aqueous dispersed citrate-coated nanostructures (adsorbents) and Cr^6+^ aqueous solution were mixed and incubated for 6 h at pH 7 at room temperature. Both citrate-capped Fe_3_O_4_ NPs and Mn_3_O_4_ NPs were used as the control group, and 0.01 M of trisodium citrate served as a background. The amounts of adsorbed Cr^6+^ per unit mass of adsorbent (*Q*_e_; mg g^−1^) were calculated using eqn (1):1
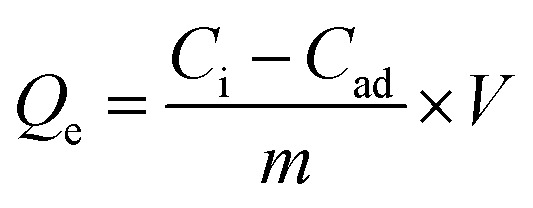
*C*_i_ and *C*_ad_ were the initial concentration of Cr^6+^, which was equal to 30 mg L^−1^, and the concentration of Cr^6+^ in the solution at the equilibrium, respectively. The total volume of the reactants mixture (*V*) was 2 mL, and the mass of adsorbents (*m*) was represented in g with respect to Mn mass fractions. The concentration of Cr^6+^ was quantified by measuring the optical density of the colour generated by the Cr^3+^ DPC (diphenylcarbazide) complex method at *λ* 545 nm.^106,107^

The adsorption isotherms of Cr^6+^ by the selected Mn_0.2_^2+^Fe_2.8_^3+^O_4_ NPs were studied due to their superior *Q*_e_. The isotherm was measured at room temperature by varying the initial Cr^6+^ concentration from 10–250 mg L^−1^ at pH 7 for 6 h contact time. The concentration of the adsorbents was adjusted to 1 mg mL^−1^. For Mn_0.2_^2+^Fe_2.8_^3+^O_4_, the mass of the adsorbent was calculated in respect to both Fe and Mn fractions which were 0.68 and 0.05, respectively. Adsorption isotherms were fitted by both Langmuir and Freundlich models.^108^

#### 3.2.7 Bacteria identification

The genome DNA of bacteria was extracted by boiling a single colony in ultra-pure water for 10 min at 95 °C. To deliver the highest DNA yield from the tested colony, FastDNA Spin Kit for Soil was applied to the boiled broth following the manufacturer's instructions. Using a Qubit 3.0 fluorometer (Life Technologies, UK), the quantity of DNA was evaluated using Qubit dsDNA broad range (2 to 1000 ng) assay kit from Invitrogen (UK). 16S rRNA genes of all strains were amplified by polymerase chain reaction (PCR) using pair primers; 1369F and 1492R primers and Luna Universal qPCR Master Mix.^109^ Using thermocycler (Cole-Parmer), cycling conditions included initial denaturation at 94 °C for 5 min, followed by 30 cycles of denaturation at 94 °C for 45 s, heating at 52 °C for 45 s and extension at 72 °C for 90 s. The final extension was tested at 72 °C for 90 s. Both the purification of amplified PCR products and Sanger sequencing were implemented using the commercial service of Source Bioscience, Cambridge, UK. The sequenced data were assigned for matching identity for species with the highest fitting 96–100% by nucleotide BLAST (Basic Local Alignment Search Tool) from the National Centre for Biotechnology Information (NCBI) database (https://blast.ncbi.nlm.nih.gov).

#### 3.2.8 Minimum inhibition concentration of Cr^6+^

To assess the impact of Cr^6+^ on the viability of the tested *Shewanella*, a Guava easyCyte® flow cytometer (Merck, UK) was used. 10 μL of homogeneous bacterial cell suspensions with OD measured at the wavelength of 600 nm equal to 0.1 was added to 80 μL of M9 minimal salts (2×) medium.^110^ This medium was supplemented by 20 mM sodium lactate as a sole electron source, 5 mL L^−1^ each of vitamins and minerals and pH was adjusted to 7.2 by 10 mM HEPES buffer.^111^ The viability of cells was counted in response to serial dilutions of Cr^6+^ (1 to 100 mg L^−1^) as a terminal electron source alone. Sodium fumarate (20 mM) was used as an alternative terminal electron acceptor to Cr^6+^. In all cases, media were purged with nitrogen gas for 5 min after bacterial inoculation. The proportion of live cells was quantified in relation to the total number of cells *via* the Live Dead BacLight Bacterial viability assay. Populations of living and/or dead bacteria were gated according to fluorescence minus one (FMO) controls using single stains of SYTO9 and propidium iodide (PI).^112^ All data are expressed as means ± standard deviation. The MIC of any agent was defined as its lowest concentration that inhibits the growth of bacteria after overnight incubation.

#### 3.2.9 Effect of the Mn_0.2_^2+^Fe_2.8_^3+^O_4_ NPs on the bio-removal of Cr^6+^

Measuring the impact of the tested NPs on the bio-removal of Cr^6+^ efficiency was tested at 50 mg L^−1^ as a sub-lethal dose of Cr^6+^ for both *S. oneidensis* and *S. loihica* while using 10 and 1 mg L^−1^ of Cr^6+^ for the biological controls (*S. oneidensis* JG1486 and JG3355, respectively). The concentration of citrate was 0.1 mM, and citrate-coated Fe_3_O_4_, Mn_0.2_^2+^Fe_2.8_^3+^O_4_ NPs, MnFe_2_O_4_ NFs and Mn_3_O_4_ NPs were adjusted to be in the range of 1 mg mL^−1^. The remaining soluble Cr^6+^ in the supernatant (Cr_s_) was filtered through a 0.45 μm membrane and quantified using Cr^3+^–DPC complex method.^106,107^ The percentage of Cr^6+^ bio-reduction was calculated in relation to the initial concentration (Cr_i_) of Cr^6+^ following eqn (2):^26,81^2
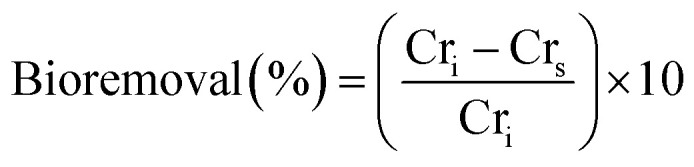


## 4. Conclusions

Adsorption of hexavalent chromium (Cr^6+^) on manganese ferrite (Mn_*x*_Fe_3−*x*_O_4_) nanostructures enhanced the bio-detoxification of Cr^6+^ by *S. oneidensis* MR-1. A synthetic platform for achieving the most suitable chemical structure of Mn_*x*_Fe_3−*x*_O_4_ nanoparticles (NPs) and nanoflowers (NFs) acting as Cr^6+^ adsorption agents was presented. At 250 °C, both divalent or trivalent manganese precursors formed spherical NPs, whereas, at 200 °C, nanoflowers were obtained using a trivalent precursor. Mn_0.2_^2+^Fe_2.8_^3+^O_4_ NPs that were prepared from divalent manganese precursor showed the highest Cr^6+^ adsorption capacity (16.8 ± 1.6 mg g^−1^) and led to 3.3 times improvement in the viability of *S. oneidensis* MR-1 in the presence of Cr^6+^ and 2.66 times an enhancement in Cr^6+^ bio-detoxification. This will open up a new venue of research using nanomaterials for boosting the bio-reduction of Cr^6+^ using bacteria.

## Author contributions

N. T. K. T. and L. C. devised and coordinated the project and provided resources. D. S. R. designed and did most of the experiments and wrote the manuscript. I. T. assisted in particle synthesis and data analysis. S. A., P. W. and M. C. helped with the microbiology work. N. T. K. T. and S. M. provided expertise, corrected the manuscript and helped to acquire funding. K. S., A. M. and D. K. carried out XPS characterization, processed data and corrected the manuscript. G. V. provided resources for characterization. L. B. helped to acquire funding.

## Conflicts of interest

The authors declare no competing financial interest.

## Supplementary Material

NA-005-D2NA00691J-s001
