# Correction: Enhanced detoxification of Cr^6+^ by *Shewanella oneidensis via* adsorption on spherical and flower-like manganese ferrite nanostructures

**DOI:** 10.1039/d3na90043f

**Published:** 2023-05-16

**Authors:** Diana S. Raie, Ioannis Tsonas, Melisa Canales, Stefanos Mourdikoudis, Konstantinos Simeonidis, Antonios Makridis, Dimitrios Karfaridis, Shanom Ali, Georgios Vourlias, Peter Wilson, Laurent Bozec, Lena Ciric, Nguyen Thi Kim Thanh

**Affiliations:** a Biophysics Group, Department of Physics and Astronomy, University College London Gower Street London UK ntk.thanh@ucl.ac.uk http://www.ntk-thanh.co.uk; b UCL Healthcare Biomagnetics and Nanomaterials Laboratories 21 Albemarle Street London UK; c UCL Electronic and Electrical Engineering, UCL Gower Street London UK; d Healthy Infrastructure Research Group, Department of Civil, Environmental & Geomatic Engineering, UCL Gower Street London UK; e Department of Physics, Aristotle University of Thessaloniki 54124 Thessaloniki Greece; f Environmental Research Laboratory, ClinicalMicrobiology and Virology, University College London Hospitals NHS Foundation Trust London UK; g Faculty of Dentistry, University of Toronto Toronto Ontario Canada

## Abstract

Correction for ‘Enhanced detoxification of Cr^6+^ by *Shewanella oneidensis via* adsorption on spherical and flower-like manganese ferrite nanostructures’ by Diana S. Raie *et al.*, *Nanoscale Adv.*, 2023, https://doi.org/10.1039/d2na00691j.

The authors regret that ref. 6, 10, 15, 20, 21, 26, 27, 43, 52, 67, 83, 84, 92, 93, 102, 111 and 112 were either incomplete or incorrect in the original manuscript. The correct references are given as ref. 1–17 below.

In addition, the author Antonios Makridis's name was incorrectly spelt as Antonis Makridis in the original manuscript. The correct name is listed above.

The Royal Society of Chemistry apologises for these errors and any consequent inconvenience to authors and readers.

## Supplementary Material
