# Supplementary material for: Enhanced detoxification of Cr6+ by Shewanella oneidensis via adsorption on spherical and flower-like manganese ferrite nanostructures
Source: Nanoscale Adv. 2023 Jan 16;5(11):2897–910. doi: 10.1039/d2na00691j (PMC10228370; doi:10.1039/d2na00691j)
Supplement: NA-005-D2NA00691J-s001 [file NA-005-D2NA00691J-s001.pdf]

Electronic Supplementary Material (ESI) for Nanoscale Advances.
This journal is © The Royal Society of Chemistry 2023

Enhanced detoxification of Cr^{6+} by *Shewanella oneidensis* via adsorption on spherical and flower-like manganese ferrite nanostructures

Diana S. Raie,[a,b] Ioannis Tsonas,[c] Melisa Canales,[d] Stefanos Mourdikoudis,[a,b] Konstantinos Simeonidis,[e] Antonis Makridis,[e] Dimitrios Karfaridis,[e] Shanom Ali,[f] Georgios Vourlias,[e] Peter Wilson,[f] Laurent Bozec,[g] Lena Ciric,[d] Nguyen Thi Kim Thanh[a,b*]

[a] Biophysics Group, Department of Physics and Astronomy, University College London, Gower Street, London, WC1E 6BT, UK

[b] UCL Healthcare Biomagnetics and Nanomaterials Laboratories, 21 Albemarle Street, London, W1S 4BS, UK

[c] UCL Electronic and Electrical Engineering, UCL, Gower Street, London, WC1E 7JE, UK

[d] Healthy Infrastructure Research Group, Department of Civil, Environmental & Geomatic Engineering, UCL, Gower Street, London, WC1E 6BT, UK

[e] Department of Physics, Aristotle University of Thessaloniki, 54124 Thessaloniki, Greece

[f] Clinical Microbiology and Virology, University College London Hospitals NHS Foundation Trust, London, UK

[g] Faculty of Dentistry, University of Toronto, Toronto, Ontario, Canada

Contents

1. Characterization of nanoparticles (NPs) 3
2. Functionalization of NPs and nanoflowers (NFs) 11
3. Raman Spectra of the selected NPs 14
4. The oxidation state of Mn and Fe in the prepared NPs 15
5. Biological studies 17

1. Characterization of nanoparticles (NPs)

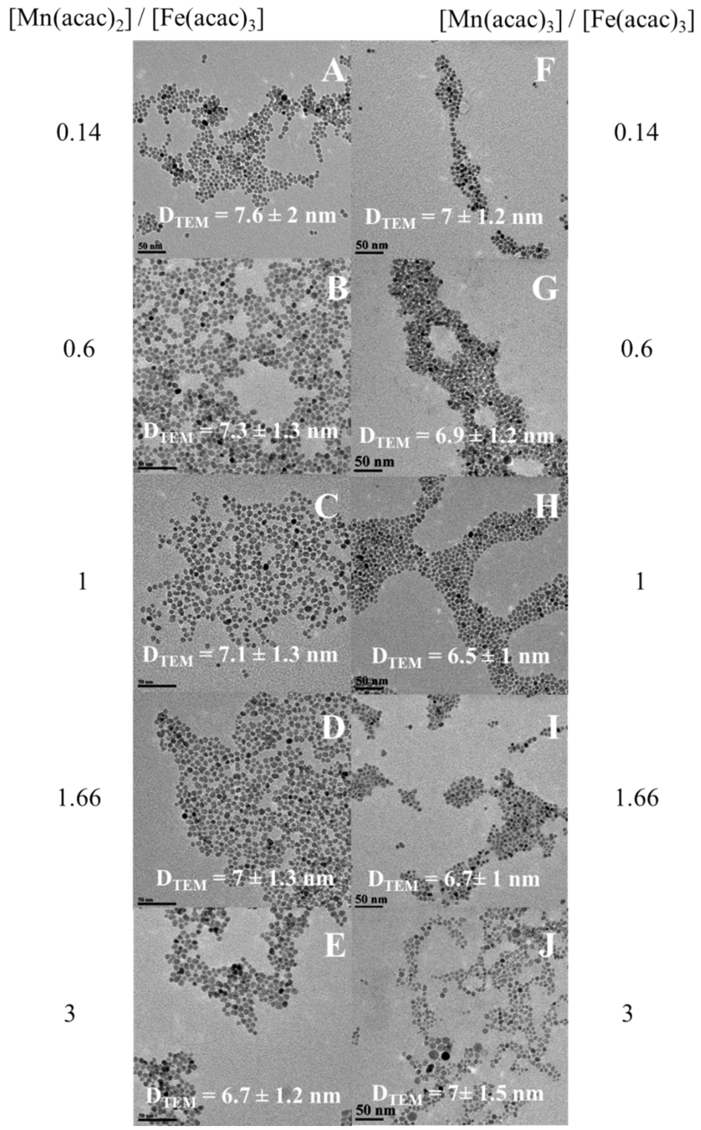

Figure S1: TEM images of spherical $Mn_xFe_{3-x}O_4$ nanoparticles (NPs) prepared by solvothermal method at 250 °C. The precursor concentration ratio of (A-E) [Mn(acac)$_2$] / [Fe(acac)$_3$] and (F-J) [Mn(acac)$_3$] / [Fe(acac)$_3$] respectively were 0.14, 0.6, 1, 1.66 and 3.

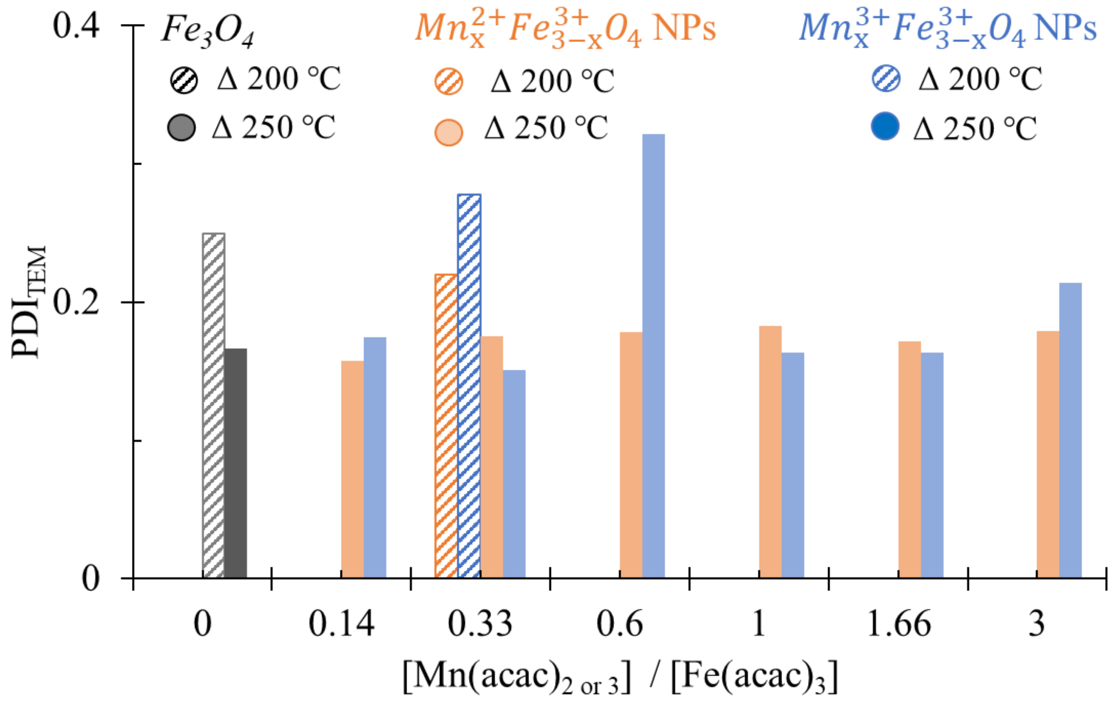

Figure S2: Polydispersity index (PDI) of NPs diameters varied with precursor concentration ratios calculated from TEM at annealing temperature (200 vs 250 °C).

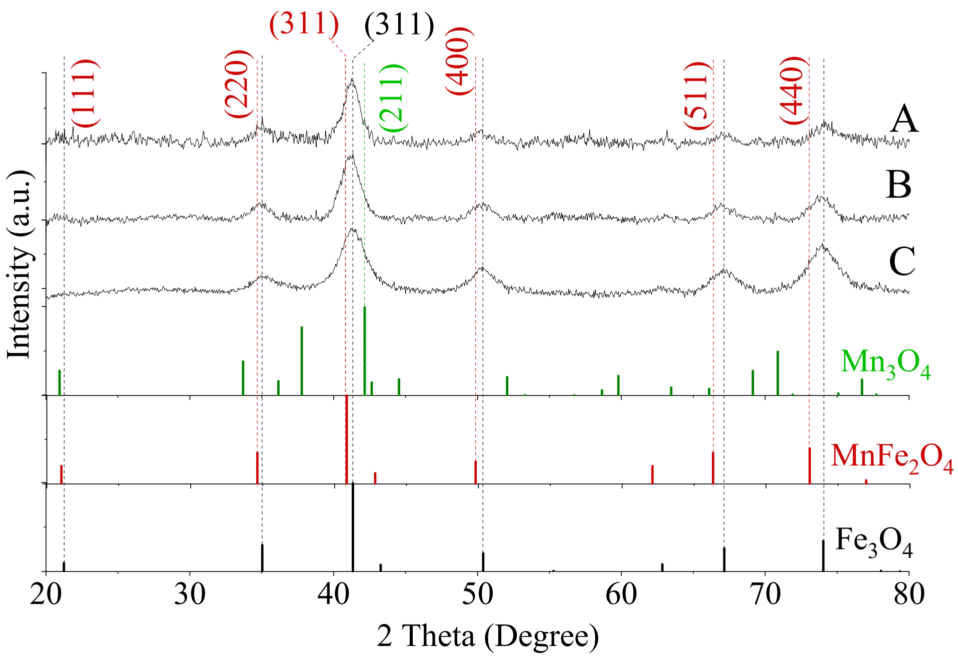

Figure S3: XRD patterns of NPs prepared at 200 °C where (A) undoped Fe_3O_4 NPs, (B) [Mn(acac)$_2$] / [Fe(acac)$_3$] was 0.33, (C) [Mn(acac)$_3$] / [Fe(acac)$_3$] was 0.33. The reference ICDD were Mn_3O_4 (PDF card no. 01-080-0382), $MnFe_2O_4$ (PDF card no 00-010-0319) and Fe_3O_4 (PDF card no 01-089-0688).

Table S1: Summary of 311 peak positions and crystal size (D_{XRD})[a] determined by XRD of $Mn_xFe_{3-x}O_4$ NPs

$[Mn(acac)_{2\ or\ 3}] / [Fe(acac)_3]$	$Mn(acac)_2$		$Mn(acac)_3$	
	2θ (311)	D_{XRD} (nm)	2θ (311)	D_{XRD} (nm)
0.14	41.33	7 ± 1	41.01	7 ± 1
0.33	41.4	7 ± 0.5	41.06	6 ± 1*
0.6	41	7 ± 1	41.07	7.5 ± 1
1	41.1	7 ± 1	41.01	6.5 ± 1*
1.66	41	6.5 ± 1*	40.92	5 ± 1**
3	41	6.5 ± 1.5*	40.87	5.5 ± 1.5**
7	-	-	-	-

*[a]D_{XRD} was presented as (mean ± standard deviation) of 3 independent synthesis experiments and *$p < 0.05$ and **$p < 0.01$ showed the statistical confidence levels by comparing the crystal size with Fe_3O_4 NPs (9 ± 1.3 nm) synthesized in the same conditions.*

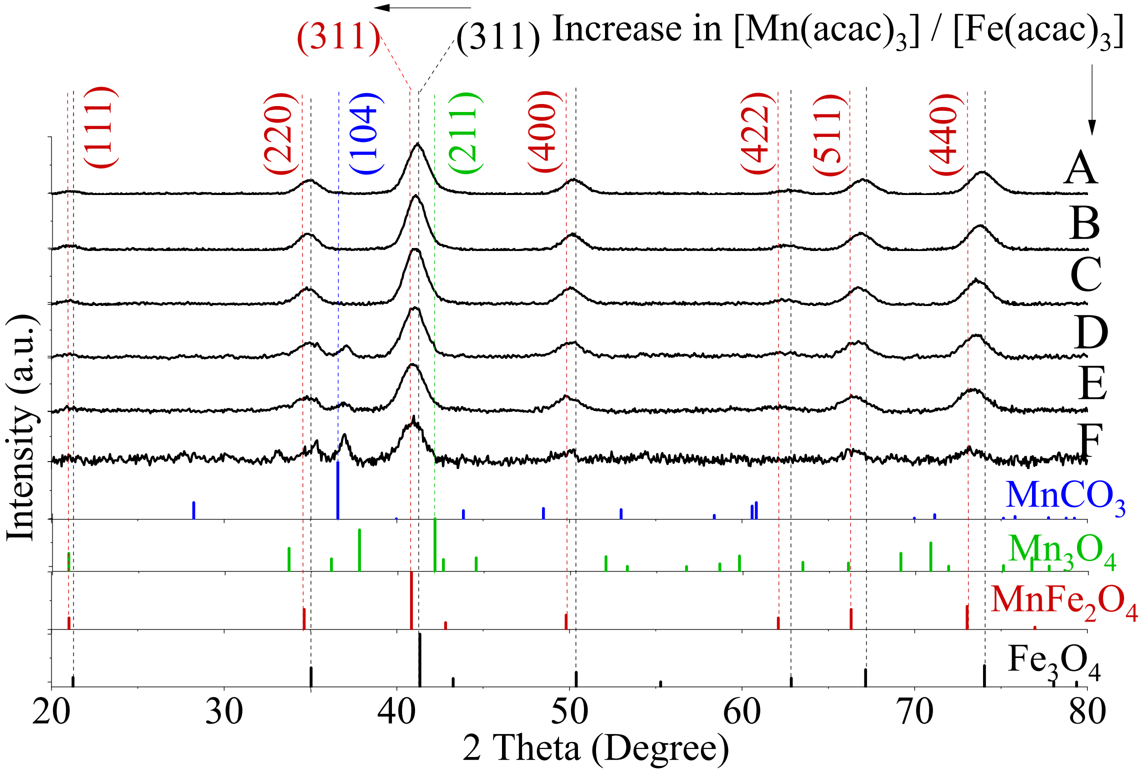

Figure S4: XRD patterns of $Mn_xFe_{3-x}O_4$ NPs where $[Mn(acac)_3]$ / $[Fe(acac)_3]$ were (A) 0.14, (B) 0.33, (C) 0.6, (D) 1 (E) 1.66 (F) 3. The reference ICDD were Mn_3O_4 (PDF card no. 01-080-0382), $MnFe_2O_4$ (PDF card no 00-010-0319) and Fe_3O_4 (PDF card no 01-089-0688). A secondary phase appears to be present for NPs prepared from $[1 \leq [Mn(acac)_{2\ or\ 3}] / [Fe(acac)_3] \leq 3]$ (D-F) which was identified as $MnCO_3$ (Reference ICDD PDF card no. 00-044-1472). Perpendicular and horizontal arrows indicated the gradual increase in $[Mn(acac)_3]$ / $[Fe(acac)_3]$ from (A-F) and the shifting in the peak of 311 from the reference Fe_3O_4 towards lower diffraction angle of $MnFe_2O_4$, respectively.

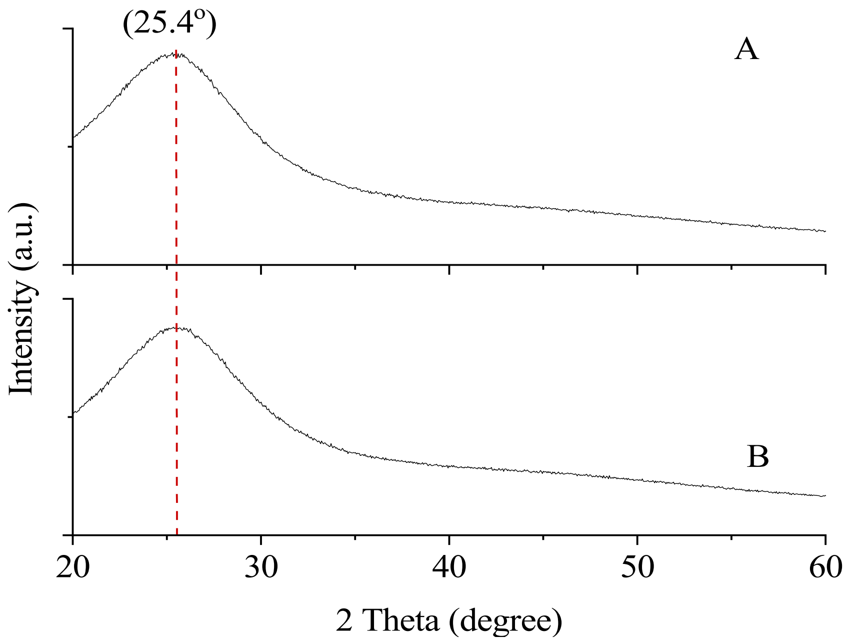

Figure S5: XRD patterns of TEG (A) before and (B) after thermal treatment at 250 °C for 6 h in autoclave.

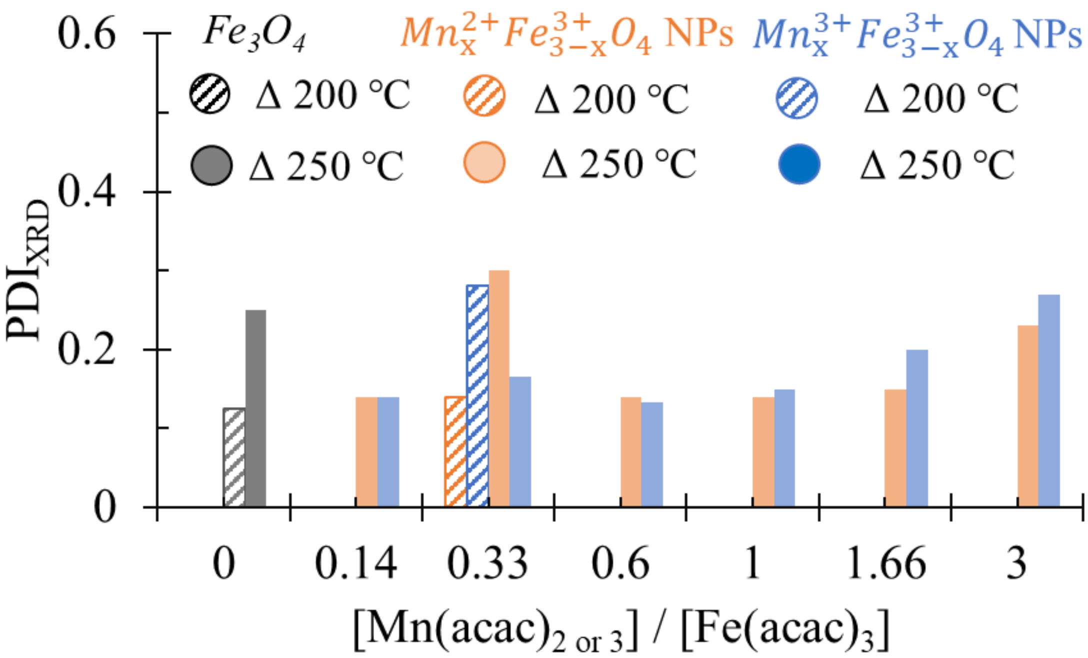

Figure S6: Polydispersity index (PDI) of NP diameters varied with precursor concentration ratios calculated from XRD at the most intense peak of (311) at annealing temperature (200 vs 250 °C).

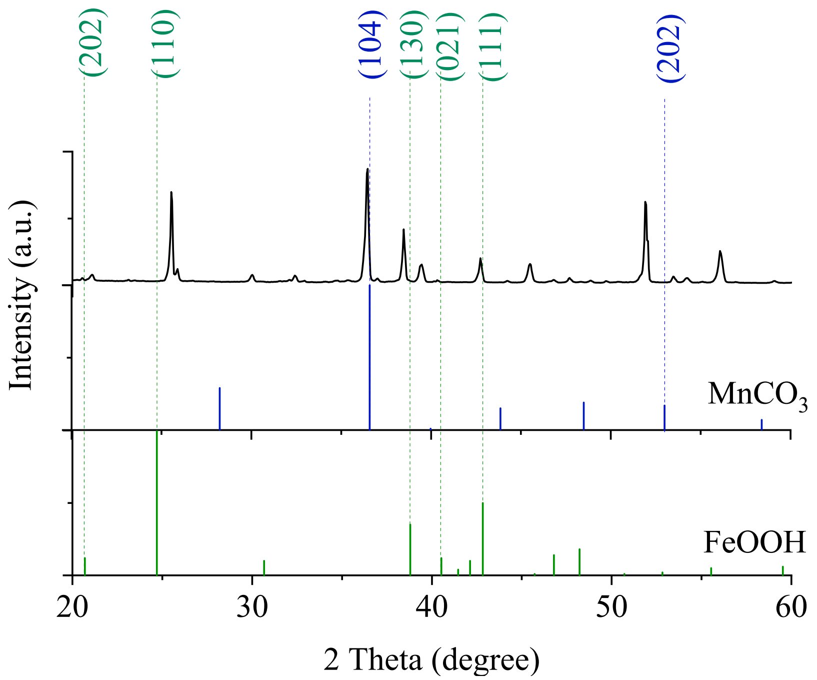

Figure S7: XRD patterns of produced sample where [Mn(acac)$_2$] / [Fe(acac)$_3$] was 7. Reference JCPDS card no. 00-044-1472, 00-029-0713, for $MnCO_3$, FeOOH, respectively.

When [Mn(acac)$_2$] / [Fe(acac)$_3$] was 7, the observed XRD peaks of the solvothermal-produced material at 250 °C cannot be assigned to well-established crystal phases of ferrite materials (Figure S7). Hence, this sample was not selected for further analysis since it could not offer critical insights on metal substitution using the above precursors.

2. **Functionalization of NPs and nanoflowers (NFs)**

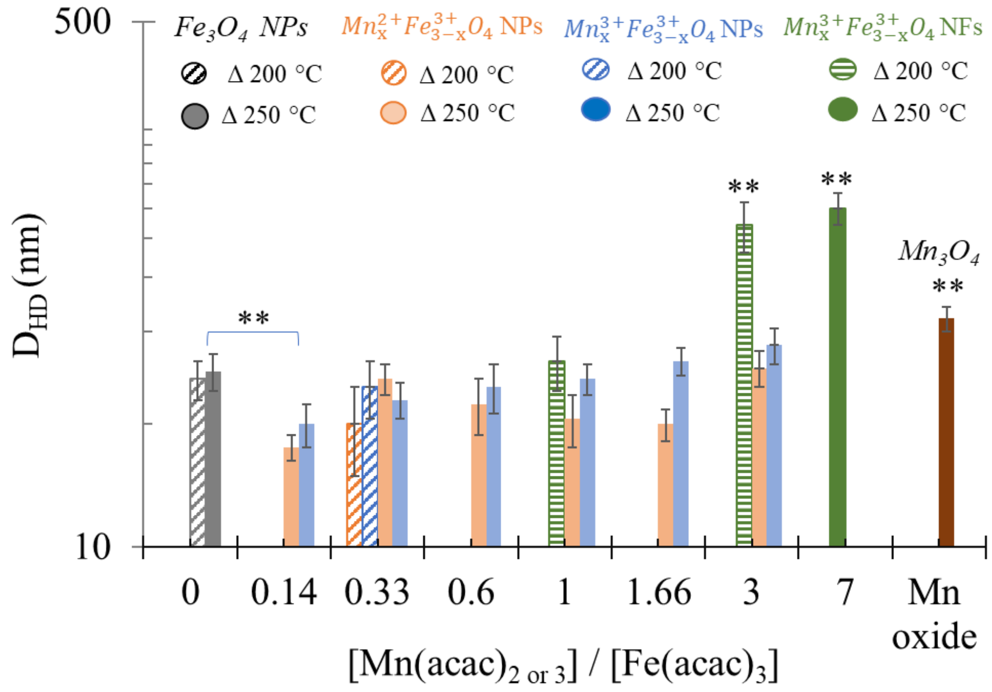

*Figure S8: Impact of variation in ratios between $[Mn(acac)_{2\ or\ 3}] / [Fe(acac)_3]$ in $Mn_xFe_{3-x}O_4$ nanostructures and synthesis temperature (200 vs 250 °C) on hydrodynamic size (D_{HD}) of NPs and NFs coated by citrate. Fe_3O_4 NPs and Mn_3O_4 NPs were represented by grey and brown bars, respectively. **$p < 0.01$ showed the statistical confidence levels when comparing the size with Fe_3O_4 NPs synthesized at 250 °C.*

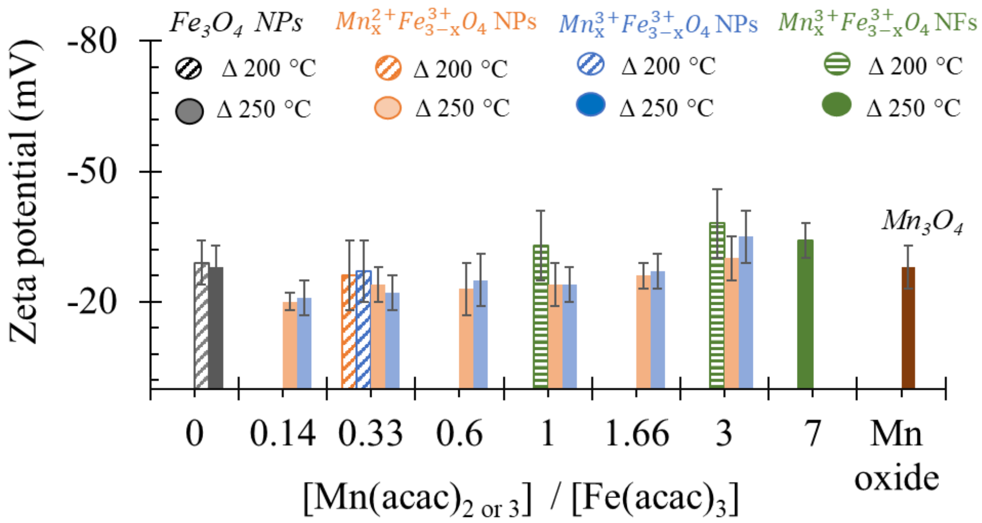

Figure S9: Impact of variation in [Mn(acac)$_{2\ or\ 3}$] / [Fe(acac)$_3$] and synthesis temperature (200 vs 250 °C) on ζ-potential of NPs and NFs coated by citrate.

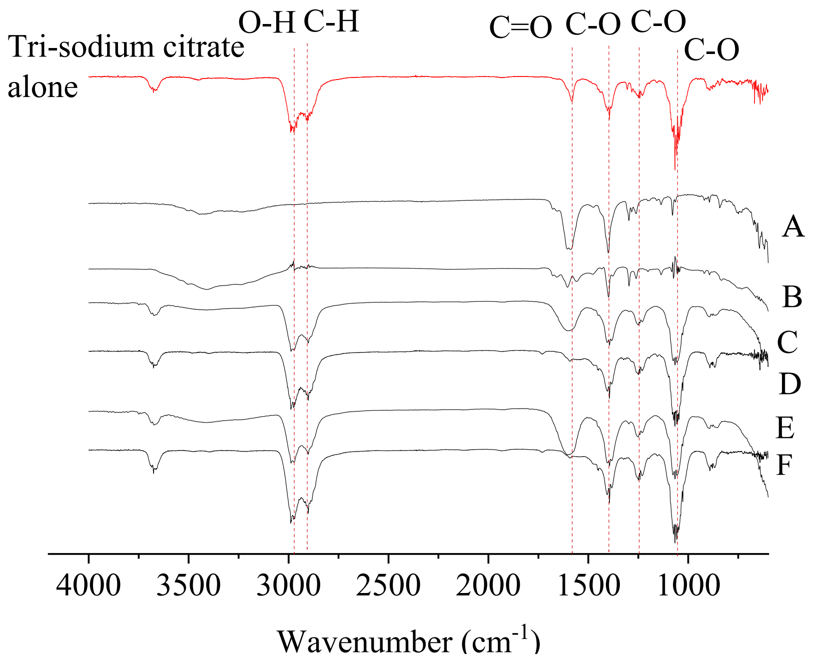

Figure S10: FTIR spectra of tri-sodium citrate alone, (A) Fe_3O_4 NPs prepared at 250 °C for 6h of synthesis time, $Mn_xFe_{3-x}O_4$ NPs prepared at 250 °C where (B) [$Mn(acac)_2$] / [$Fe(acac)_3$] and (C) [$Mn(acac)_3$] / [$Fe(acac)_3$] were 0.33, for 6h of synthesis time (D) [$Mn(acac)_3$] / [$Fe(acac)_3$] was 3, (E) $MnFe_2O_4$ NFs [$Mn(acac)_3$] / [$Fe(acac)_3$] was 3 and (F) Mn_3O_4 NPs. and all samples were coated by citrate.

3. Raman Spectra of the selected NPs

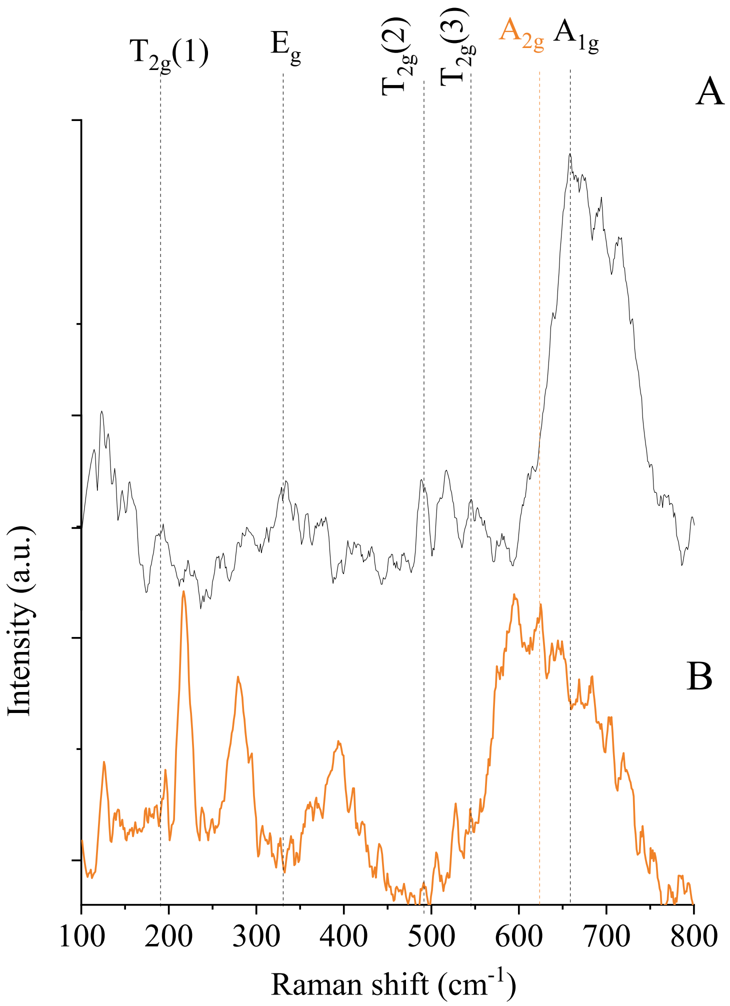

Figure S11: Raman spectra of (A) Fe_3O_4 NPs, (B) $Mn^{2+}_{0.2}Fe^{3+}_{2.8}O_4$ NPs.

4. **The oxidation state of Mn and Fe in the prepared NPs**

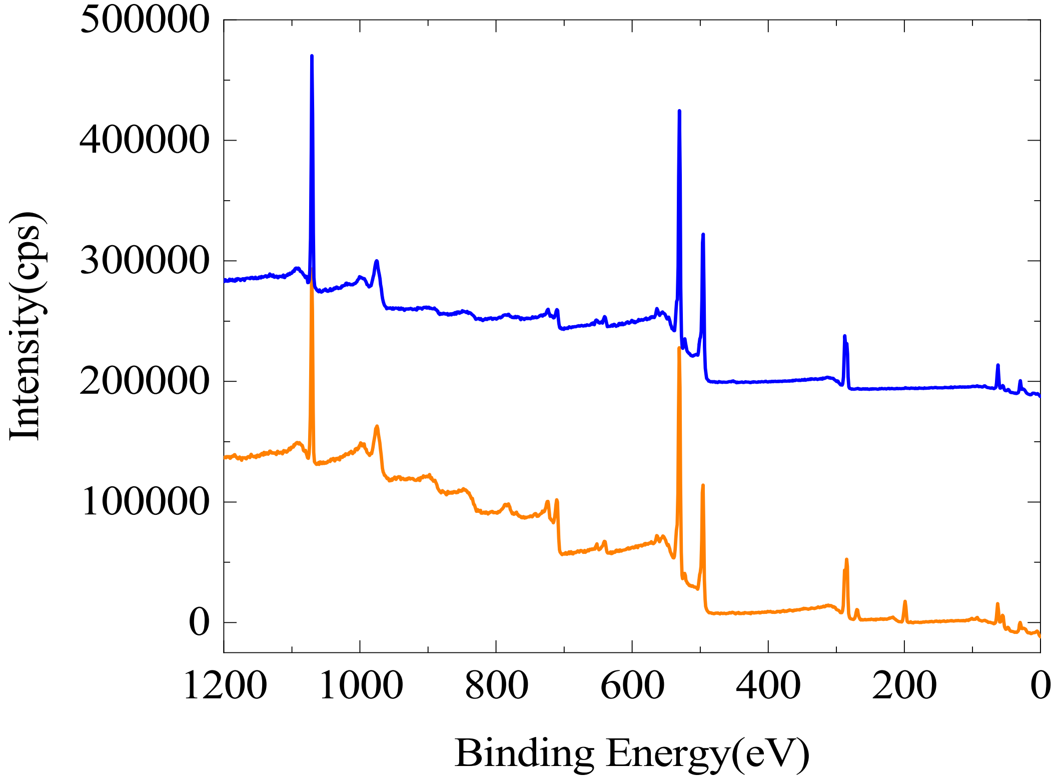

Figure S12: XPS spectra of $Mn_xFe_{3-x}O_4$ NPs where orange and blue represent $Mn^{2+}_{0.2}Fe^{3+}_{2.8}O_4$ and $Mn^{3+}_{x}Fe^{3+}_{3-x}O_4$ respectively.

Table S2: Peak positions and full width at half maximum (FWHM) of the XPS of Mn 2p peak for Mn^{x+}.

	$Mn^{2+}_{0.2}Fe^{3+}_{2.8}O_4$					$Mn^{3+}_{x}Fe^{3+}_{3-x}O_4$			
Binding Energy $(BE)_{Mn}$ (eV)	640	642	645	651	653	640	642	651	653
FWHM	2.7	2.1	2.9	2.5	1.9	2.2	2.25	2.7	3
Peak weighting (%)	22.3	10.2	14	20.7	15.7	21.7	22.1	26.6	29.6

Table S3: Peak positions and FWHM of the XPS of Fe 2p peak for Fe^{x+}.

	$Mn^{2+}_{0.2}Fe^{3+}_{2.8}O_4$						$Mn^{3+}_{x}Fe^{3+}_{3-x}O_4$				
BE_{Fe} (eV)	710	712	718	723	726	732	710	713	718	723	726
FWHM	2.5	3.3	6.5	3.2	4.6	3.6	2.7	2.8	5.7	4	5.4
Peak weighting (%)	10.5	14	27.4	13.5	19.4	15.2	13.1	13.6	27.7	19.4	26.2

5. **Biological studies**

Table S4: Molecular identification of the tested bacteria

Bacterial strain	16S rRNA gene sequence	*BLAST bacterial matches at NCBI database*
S. oneidensis MR-1	AAGNGTGAGCGCCCCCCGAAGGTTAAGCTACCCACTTCTTTTGCAGCCCACTCCCATGGTGTGACGGGCGGGTGTGTACAAGGCCCGGGAACGTATTCACCGA	CP053946.1, 98.98 %
S. loihica PV-4	GGTGAGCGCCCCCCGAAGGTTAAGCTACCCACTTCTTTTGCAGCCCACTCCCATGGTGTGACGGGCGGTGTGTACAAGGCCCGGGAACGTATTCACCGAA	CP053946.1, 100 %
S. oneidensis JG1486	TTGCAAAAGAAGCTAGNTAGCCTTAACCTTCGGGAGGGCGCTTACCACTTTGTGATTCATGACTGGGGTGAAGTCGTAACAAGGTAAC	EU567031.1, 96.6 %
S. oneidensis JG3355	CACTTAGGCGGCTGGCTCCAAAGGTTACCTCACCGACTTCGGGTGTTACAAACTCTCGTGGTGTGACGGGCGGTGTGTACAAGGCCCGGGAACGTATTCACCGA	KT336053.1, 99.99 %
